# Genes of the Ubiquitin Proteasome System Qualify as Differential Markers in Malignant Glioma of Astrocytic and Oligodendroglial Origin

**DOI:** 10.1007/s10571-022-01261-0

**Published:** 2022-07-27

**Authors:** Jerry Vriend, Thomas Klonisch

**Affiliations:** Department of Human Anatomy and Cell Science, Max Rady College of Medicine, Max Rady Faculty of Health Sciences, University of Manitoba, Rm34, BMSB, 745 Bannatyne Ave, Winnipeg, MB R3E0J9 Canada

**Keywords:** Glioblastoma, Ubiquitin conjugase, Ubiquitin ligase, CDC20, Immunoproteasome

## Abstract

We have mined public genomic datasets to identify genes coding for components of the ubiquitin proteasome system (UPS) that may qualify as potential diagnostic and therapeutic targets in the three major glioma types, astrocytoma (AS), glioblastoma (GBM), and oligodendroglioma (ODG). In the Sun dataset of glioma (GEO ID: GSE4290), expression of the genes *UBE2S* and *UBE2C*, which encode ubiquitin conjugases important for cell-cycle progression, distinguished GBM from AS and ODG. KEGG analysis showed that among the ubiquitin E3 ligase genes differentially expressed, the Notch pathway was significantly over-represented, whereas among the E3 ligase adaptor genes the Hippo pathway was over-represented. We provide evidence that the UPS gene contributions to the Notch and Hippo pathway signatures are related to stem cell pathways and can distinguish GBM from AS and ODG. In the Sun dataset, *AURKA* and *TPX2,* two cell-cycle genes coding for E3 ligases, and the cell-cycle gene coding for the E3 adaptor *CDC20* were upregulated in GBM. E3 ligase adaptor genes differentially expressed were also over-represented for the Hippo pathway and were able to distinguish classic, mesenchymal, and proneural subtypes of GBM. Also over-expressed in GBM were *PSMB8* and *PSMB9*, genes encoding subunits of the immunoproteasome. Our transcriptome analysis provides a strong rationale for UPS members as attractive therapeutic targets for the development of more effective treatment strategies in malignant glioma.

**Graphical abstract:**

Ubiquitin proteasome system and glioblastoma:

E1—ubiquitin-activating enzyme, E2—ubiquitin-conjugating enzyme, E3—ubiquitin ligase. Ubiquitinated substrates of E3 ligases may be degraded by the proteasome. Expression of genes for specific E2 conjugases, E3 ligases, and genes for proteasome subunits may serve as differential markers of subtypes of glioblastoma.

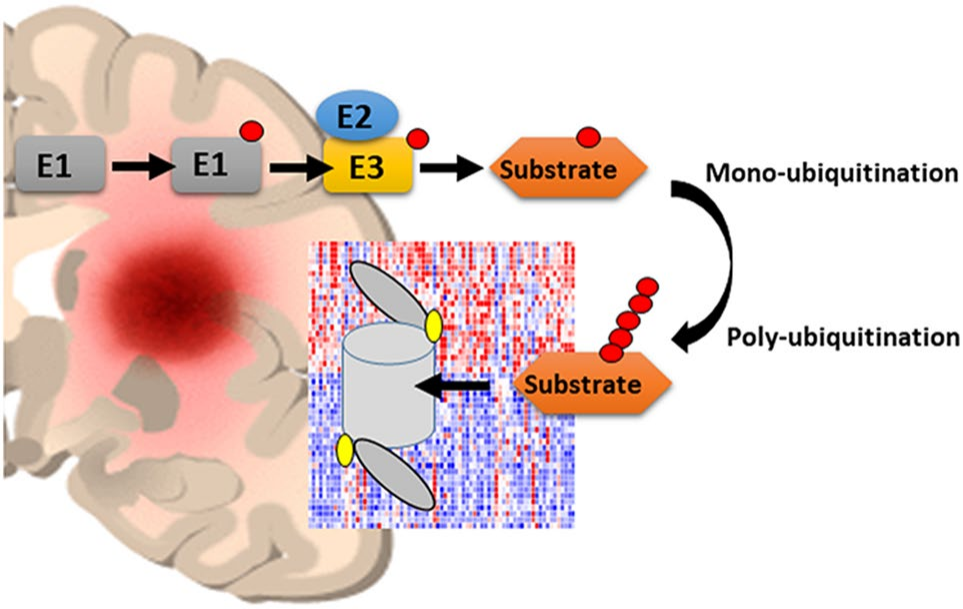

## Introduction

Gliomas derive from three main types of supporting glial cells within the brain, including astrocytes, ependymal cells, and oligodendrocytes, and account for 33% of all primary brain tumors or 78% of all malignant primary brain tumors. Astrocytomas grades I–III and glioblastoma (GBM), a grade IV highly aggressive astrocytoma, derive from astrocytes and constitute the largest group among the gliomas. Higher grade astrocytomas are found most commonly in the cerebrum of adults and GBM accounts for more than 50% of all gliomas. Oligodendrogliomas (ODG) originate mainly in younger adults from oncogenic oligodendrocytic progenitors, account for 2–4% of all primary brain tumors, and have a better prognosis than most other malignant gliomas. Typically, ODG carry IDH1 (Watanabe et al. 2009) and TERT mutations (Lee et al. 2017) and a chromosomal 1p/19q co-deletion (Lee et al. 2018). The inclusion of molecular diagnostic markers has resulted in a major reclassification of CNS tumors which is summarized in the 2021 fifth edition of the WHO classification of Tumors of the Central Nervous System (Louis et al. 2021).

The incidence of GBM is approx. 3.19/100,000 and reaches a peak at 75–84 years, with white males being affected more commonly. GBM is rare in children, constituting less than 3% of all pediatric primary brain.tumors, and, contrary to GBM in adults, children with GBM have a slightly higher 5 year survival time of 12% (Dolecek et al. 2012). Despite surgery and radio-chemotherapy, GBM in adult patients have an average survival time of 1.5 years and a 5 year survival time of < 5% (Perry et al. 2007). Contributing factors to this fatal outcome are late diagnosis, considerable heterogeneity and plasticity of GBM cells, invasiveness, development of therapeutic resistance, and fatal GBM recurrences. Pre-operative stratification of patients is challenging, with age and Karnofsky performance scale currently being the only established predictive clinical factors (Ening et al. 2015).To improve prospective stratification and better inform therapeutic strategies, additional demographic (Charlson comorbidity index) and clinical/imaging criteria (GBM size, location, MRI-based methods) have emerged (Ening et al. 2015; Thomas et al. 2013; Jain et al. 2013). Large scale gene expression analysis has identified three distinct GBM subtypes, proneural, classical, and mesenchymal, while an additional neural subtype is controversial (Verhaak et al. 2010; Phillips et al. 2006; Sidaway 2017; Piao et al. 2013). Despite distinct genetics in different GBM subtypes (Ening et al. 2015; Thomas et al. 2013; Jain et al. 2013; Ludwig and Kornblum 2017; Karsy 2015; Wick and Platten 2014), differentiation from a proneural to a mesenchymal GBM subtype occurs in drug-resistant cell lines (Piao et al. 2013) and so does the progression of lower grade AS into GBM (classical) (Ohgaki and Kleihues 2007). The genetic GBM subtype classification has had little impact on treatment options for GBM patients. The discovery of activating EGFR (Epidermal Growth Factor receptor) mutations, however, has resulted in improved treatment options for the classical GBM subtype (Hovinga et al. 2019).

Several clinical drug trials aimed at improving therapeutic outcome in GBM have failed (Gilbert et al. 2013). Thus, there is an urgent need to find additional molecular pathways for more effective therapeutic targets. We view members of the ubiquitin–proteasome system (UPS) as attractive potential therapeutic targets because UPS proteins are integral to cellular homeostasis and critically important in regulating fate and function of proteins under cellular stress in cancer cells. Cell stress from exogenous and endogenous stimuli can result in the formation of misfolded proteins which eventually are removed by the UPS or can cause cell death (Houck et al. 2012). The UPS is highly conserved among eukaryotes and active in all cells where UPS mediated post-translational protein ubiquitination involves the covalent attachment of a small 76 amino acid molecule ubiquitin (8.6 kDa) to a target protein. This is facilitated by a 3-step coordinated enzymatic cascade that is initiated by an ATP-consuming ubiquitin-activating (E1) enzyme (Schulman and Harper 2009), followed by an ubiquitin-conjugating (E2) enzymatic step (Liu et al. 2020a), and completed by a ubiquitin ligase (E3) (Medvar et al. 2016). Aiding the process are E3 ligase adaptors that serve as substrate recognition proteins (Leon and Haguenauer-Tsapis 2009). The terminal glycine residue of a single ubiquitin (mono-ubiquitination) or a poly-ubiquitin chain (poly-ubiquitination) covalently links to a lysine residue on a target protein. Covalent linkages among ubiquitins to form poly-ubiquitin chains can utilize any of the seven lysine residues of ubiquitin, or the initial methionine residue (Komander and Rape 2012). The most frequently used, K48- and K29-linked poly-ubiquitin chains, are canonical signals for 26S proteasomal degradation and endoplasmic reticulum (ER) associated degradation (ERAD) (Leto et al. 2019), whereas K63-linked poly-ubiquitin chains (and mono-ubiquitination) are the code enabling a diverse array of non-proteasomal functions, including protein translation, sorting, complex formation, and phosphorylation, as well as RNA splicing, DNA repair, endocytosis, autophagy, and transcription (Pickart 1997; Saeki et al. 2009; Wang et al. 2001; Deng et al. 2000; Doil et al. 2009; Huang et al. 2013; Lauwers et al. 2009; Song et al. 2010; Spence et al. 2000; Kodadek et al. 2006). Mono-ubiquitination of transcriptional activators, followed by poly-ubiquitination, has been reported to regulate transcription cycling in the ‘timer’ or so called ‘black widow’ models (Kodadek et al. 2006). According to these models, mono-ubiquitination facilitates the action of transcription factors, while subsequent poly-ubiquitination leads to their degradation by the proteasome. Mono-ubiquitination of H2A histone also contributes to this regulation of transcription (Zhou et al. 2008).

The UUCD database currently assigns human ubiquitin related molecules to one human ubiquitin-activating (E1) enzyme (UBA), 43 E2 ubiquitin-conjugating enzymes, 468 enzymes with E3 ligase activity, and 538 E3 ligase adaptors. This inflation of “ubiquitin writers” at the level of E2 ubiquitin conjugases and E3 ubiquitin ligases reflects increasing functional diversification to enable these ubiquitin enzymes to regulate cellular homeostasis of diverse and selected regulatory functions and signaling processes. Additionally, the ubiquitination system includes over 100 deubiquitinases (DUBs) which are implicated in all cellular processes by enzymatically removing ubiquitin groups from proteins (Clague et al. 2019). This ensures that protein ubiquitination is reversible and unbound ubiquitin is recycled for the UPS and ERAD pathways (Verma et al. 2002). The members of the seven evolutionarily conserved DUB families (USP, UCH, OTU, MJD/ Josephin, MINDY, ULP, JAMM/ MPN and the recently identify ZUP1/ ZUFSP (Kwasna et al. 2018) interact with specific substrates and show specificity for selected ubiquitin linkages (Clague et al. 2019; Ambroggio et al. 2004). The E1-3 ubiquitin enzymes, DUBs, and different proteasomal subunits are emerging as promising new drug targets in cancer treatment. Brain tumors remain challenging because of the limited ability of several UPS targeting drugs to penetrate the blood–brain barrier (BBB). Treatment failures may result from underappreciated differences in UPS gene expression profiles in different malignant gliomas and within different genetically defined GBM subtypes.

Herein, we have investigated the expression profiles of genes encoding components of the UPS in human gliomas of astrocytic (AS), GBM, and oligodendroglial origin (ODG) as well as in classic, mesenchymal and proneural GBM subtypes. Differences in the expression of selected UPS components in a specific type of malignant glioma may identify suitable therapeutic UPS targets and/or foster the design of specifically tailored drugs. Using publicly available datasets, we compared the gene expression profiles for ubiquitin activators, ubiquitin ligases (E3 ligases), ubiquitin ligase adaptors (E3 adaptors), deubiquitinases (DUBs), and genes encoding proteasome subunits to identify potential new therapeutic targets for these malignant glioma. KEGG (Kyoto Encyclopedia of Genes and Genomes) identified main pathways associated with differentially expressed UPS genes in subsets of gliomas and GBM subtypes. Our analysis of E3 ligases and adaptors in GBM found a strong association with the NOTCH and HIPPO pathways. While the expression of NOTCH1 and its ligands affect glioma proliferation (Purow et al. 2005), the role of NOTCH signaling in glioma development appears context dependent and is incompletely understood (Parmigiani et al. 2020). This may explain contradictory reports on the NOTCH system either promoting or suppressing glioma progression (Parmigiani et al. 2020). The HIPPO signaling pathway is important in various cancers and transcription of Hippo pathway genes are reported to form a set of potential tumor markers (Wang et al. 2018). KEGG pathway analysis in the gene ontology categories of stem cell differentiation and stem cell proliferation identified UPS genes that were differentially expressed in malignant glioma of astrocytic and oligodendroglial origin.

## Methods

We analyzed two public gene expression datasets through R2: Genomic analysis and visualization platform (http://r2.amc.nl). The Sun glioma dataset (GEO ID: GSE4290; R2 ID: Sun Mixed Brain Glioma 180) was used to compare expression of genes encoding UPS associated factors in 153 tumor samples classified as AS (*n* = 26, all grade 2 or 3), GBM (*n* = 77, grade 4), ODG (*n* = 50) and non-tumor (NT) brain tissue obtained from epilepsy patients (*n* = 23) (Sun et al. 2006). Gene expression data from the non-tumor brain tissues had been added subsequently to the original Sun dataset (Tumor – glioma, GSE4290). We employed the appended Sun mixed glioma dataset (Tumor – mixed glioma) with the NT brain tissue data to create figures depicting differential expression of selected genes. The TCGA (Tumor Cancer Genomic Atlas) glioblastoma dataset (R2 ID: Tumor Glioblastoma TCGA 540) was used to compare expression of UPS associated genes in 85 GBM samples classified as classical (*n* = 17), mesenchymal (*n* = 27), neural (*n* = 17), and proneural (*n* = 24) GBM subtypes (Verhaak et al. 2010). In the present study, we excluded the neural GBM subtype and, instead, focused on the other three GBM subtypes (Sidaway 2017). The Sun glioma study used the Affymetrix gene chip U133p2, while the GBM study used the Affymetrix chip U133a.

The datasets were scanned by the Ubiquitin and Ubiquitin-like Conjugation database (UUCD) program (http://iuucd.biocuckoo.org) to identify genes for the following components of the UPS: ubiquitin activators, ubiquitin conjugases, ubiquitin ligases, adaptors for ubiquitin ligases, deubiquitinases, and proteasome subunits. Differential gene expression between the malignant gliomas studied (AS, GBM, ODG) and between the three GBM subtypes (classic, mesenchymal, and proneural) was determined by analysis of variance (ANOVA) through the R2 Genomics site. Heatmaps and hierarchical cluster analysis of each dataset (Sun gliomas and TCGA GBM subtypes) were performed with the Morpheus program (Broad Institute. Cambridge, Massachusetts). For the heatmaps a statistical difference of *p* < 1.0 × 10^*−*5^ in the ANOVA between subgroups was chosen. KEGG analysis was used to identify over-represented pathways among the UPS genes in the AS, GBM, and ODG malignant gliomas and in the GBM subtypes. KEGG analysis was also used to identify UPS genes over-represented in pathways of stem cell differentiation and proliferation, and cell-cycle regulation in the SUN and TCGA datasets.

A third dataset, available through the R2 Genomics site, the French dataset (GEO ID: GSE16022; R2 ID; Tumor Glioma French 284), was used to study Kaplan–Meier survival curves associated with selected differentially expressed genes of the Sun glioma dataset.

## Results and Discussion

### Selected E2 Conjugases, but not E1 Activator, are Upregulated in GBM

**Fig. 1:**
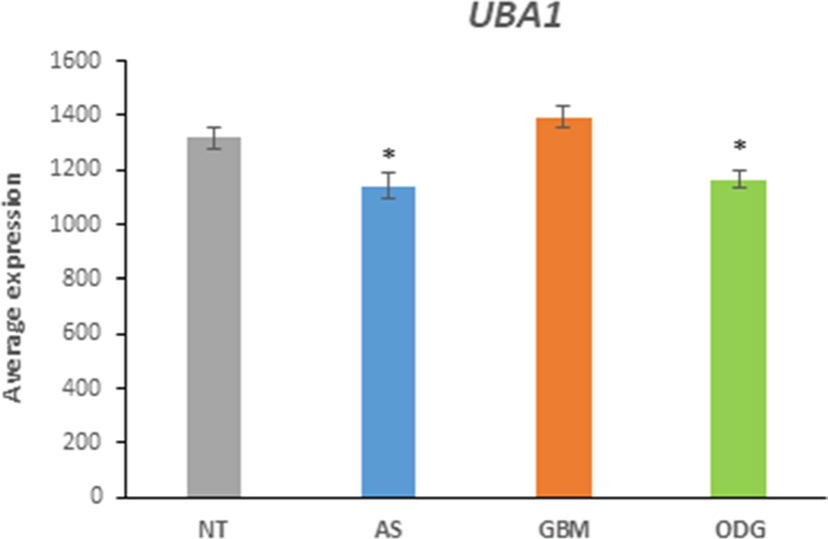
Differential expression of the gene encoding ubiquitin-activating enzyme, *UBA1*, in AS, GBM, and ODG glioma and in non-tumor (NT) brain tissues. By ANOVA, *F = *9.47, *p* = 7.99 × 10^−6^. *t test revealed that *UBA1* expression in GBM was not significantly different from NT, but AS and ODG were lower than in NT (*t* = 2.80, *p* < 0.01; *t = *2.94, *p* < 0.01)

Expression of the gene encoding the ubiquitin-activating enzyme, *UBA1*, in GBM was not significantly different from that of non-tumor tissue (NT), but AS and ODG means were significantly lower than NT, *p* < 0.01) (Fig. 1).

**Fig. 2:**
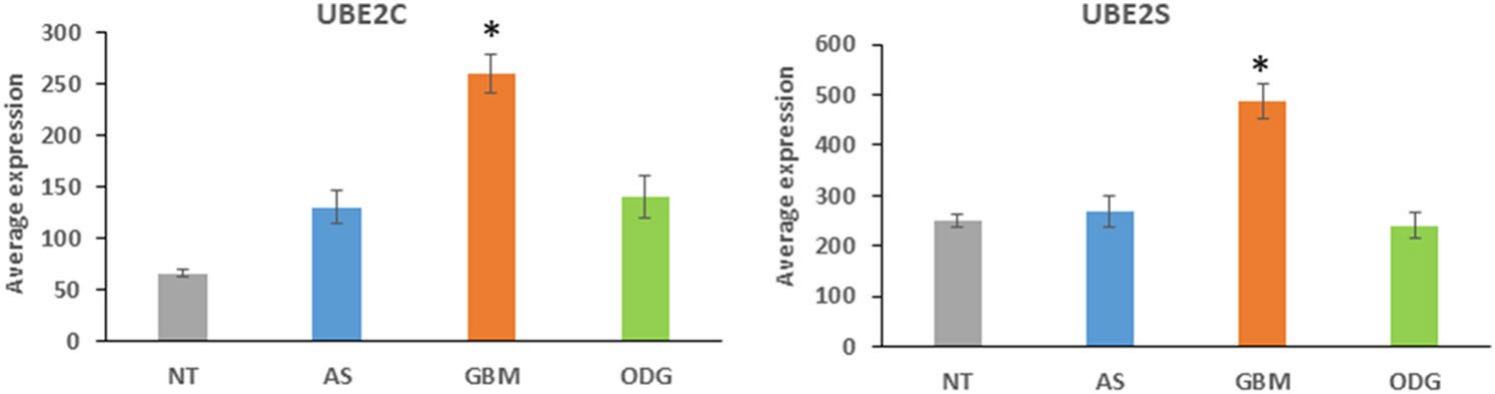
Differential expression of ubiquitin conjugase genes. ANOVA identified *UBE2C* and *UBE2S* genes upregulated in GBM samples (*UBE2C*: *F = *16.40, *p* = 2.04 × 10^−9^; *UBE2S*: *F = *15.32, *p* = 7.09 × 10^−9^). *t test showed that *UBE2C* expression was significantly greater in GBM than in all other tissue groups: (NT) (*t* = 5.62, *p* < 0.0001), AS (*t* = 3.86; *p* < 0.001), ODG (*t* = 4.19, *p* < 0.0001). *UBE2S* expression was also significantly greater in GBM than in all other tissue groups: NT (*t = *3.73, *p* < 0.001), AS (*t = *3.50, *p* < 0.001), ODG (*t = *5.22, *p* < 0.0001)

Figure 2 shows differential expression of two genes encoding ubiquitin conjugases, *UBE2C* and *UBE2S*. The association of these genes with cell-cycle regulation and the anaphase promoting complex/cyclosome (APC/c) has been well documented. APC/c is a ubiquitin ligase complex that regulates the degradation of cell-cycle proteins during mitotic exit (Manchado et al. 2010) and subsequent re-initiation of transcription of cell-cycle genes (Manchado et al. 2010). We identified a highly significant upregulation of *UBE2S* and *UBE2C* in GBM tissues versus AS, ODG, and NT brain tissue (*UBE2C*, *F = *16.397, *p* = 2.04 × 10^*−*9^; *UBE2S*, (*F = *15.315, *p* = 7.09 × 10^*−*9^). *UBE2T* was also elevated in GBM (*F = *5.684, *p* = 9.81 × 10^*−*4^) but to a lesser degree of statistical significance. *UBE2C* expression was significantly elevated when compared to the NT group by approximately fivefold (*t* = 5.62, *p* < 0.0001), whereas *UBE2S* approximately a twofold difference (*t* = 3.73, *p* < 0.001). These high statistical differences suggest a role for transcription of these genes in tumorigenesis of the GBM.

*In summary*, the data suggest that genes encoding E2 conjugases are involved in the dysregulation of cell-cycle proteins in GBM.

### Expression of E3 Ligase Genes Distinguish GBM from AS and ODG and are Associated with Survival

**Fig. 3:**
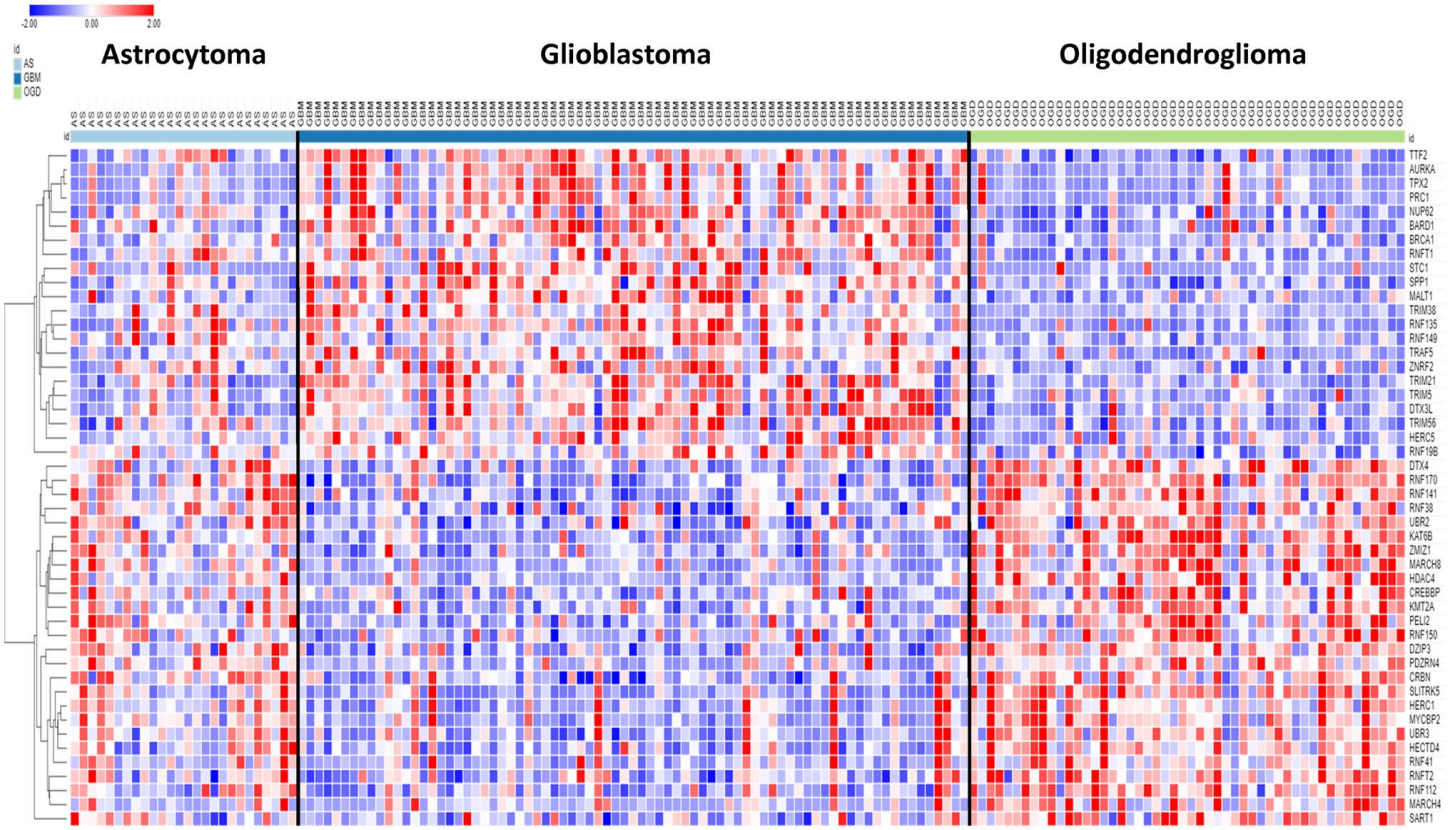
Heatmap showing relative expression of genes encoding E3 ligases in the Sun glioma dataset (48 genes differentially expressed at *p* < 1.0 × 10^−5^). High expression of the top 22 genes (red in GBM) and low expression of the bottom 26 genes (blue in GBM) was associated with lower survival in Kaplan–Meier curves derived from the French glioma dataset

A shortlist of differentially expressed genes coding for E3 ligases and E3 ligase adaptors distinguishing GBM, AS, and ODG was identified by ANOVA of log2 data using the Sun glioma dataset (Tumor – glioma) and the R2 Genomics Analysis and Visualization Platform. KEGG analysis identified pathways associated with these differentially expressed genes of the Sun dataset. A cutoff of *p* < 1.0 × 10^*−*5^ was used for inclusion in the heatmap and cluster analysis. The results of the hierarchical cluster analysis, depicted in the heatmap (Fig. 3), illustrate differential gene expression (*p* < 1.0 × 10^*−*5^) of 48 genes encoding E3 ligases in GBM, AS, and ODG glioma. E3 ligases segregated into two major clusters with gene expression either higher or lower in GBM relative to the ODG group (Fig. 3). Intermediate levels of gene expression were noted in the AS group.

To study Kaplan–Meier survival curves associated with the E3 ligase genes in the heatmap of Fig. 3, we used the glioma dataset of French (44) in the R2 genomics site. Contrary to the Sun dataset, the French glioma dataset included survival data. We identified 22 E3 ligase genes in the heatmap of Fig. 3 that were expressed higher in GBM than in ODG (red cluster in the GBM group). The Kaplan–Meier curves derived from the French dataset revealed that higher expression of each of these E3 ligase genes was associated with significantly shorter survival. Conversely, 26 of the E3 ligase genes in the heatmap of Fig. 3 were identified as having lower expression in GBM than in ODG (blue cluster in GBM group). In the French dataset, Kaplan–Meier curves showed that lower expression of each of these genes was also associated with significantly shorter survival.

The two E3 ligases genes with most significant (by ANOVA) over-expression in GBM were *TTF2* and *AURKA*. The two E3 ligase genes with most significant relative higher expression in ODG were *DTX4* and *RNF170*. Three of the TRIM genes whose expression was elevated in GBM (compared to ODG), *TRIM21*, *TRIM5*, and *TRIM38*, encode proteins that (i) regulate viral entry into the host cell, (ii) are involved in the innate immune response to viruses and (iii) respond to INF-gamma signaling with upregulated expression (Carthagena et al. 2009).

### Gene Expression for E3 Ligases and the NOTCH Signaling Pathway in Glioma

KEGG pathways over-represented by the E3 ligase genes of Fig. 3 are shown in Table 1. The main pathway over-represented in this group of genes was the Notch signaling pathway, which was shown previously to be highly active in GBM and GBM stem cells (GSC) (Bazzoni and Bentivegna 2019). The genes for E3 ligases significantly (at *p* < 1.0 × 10^*−*5^) associated with the Notch pathway included *DTX3L*, *DTX4*, *CREBBP*, and *HDAC4*. In the French dataset, high expression of *DTX3L* and low expression of *DTX4*, *CREBBP*, and *HDAC4* was associated with shorter survival (*p* = 1.8 × 10^*−*14^, *p* = 2.8 × 10^*−*4^, *p* = 2.1 × 10^*−*14^, and *p* = 1.1 × 10^*−*14^, respectively).

**Table 1:** Over-represented KEGG pathways associated with differential expression of ubiquitin E3 ligases in the Sun glioma dataset

KEGG pathway over-representation^a^	*p* for pathway	Genes
Notch signaling	3.4 × 10^*−*10^	*CREBBP, DTX3L, DTX4*
MicroRNAs in cancer	1.5 × 10^*−*3^	*BRCA1, CREBBP, HDAC4*
Viral carcinogenesis	4.6 × 10^*−*3^	*CREBBP, HDAC4, TRAF5*
Epstein-Barr virus infection	6.6 × 10^*−*^^3^	*CREBBP, HDAC4, TRAF5*

^a^Using gene expression (log 2) of genes in Fig. 3

**Fig. 4:**
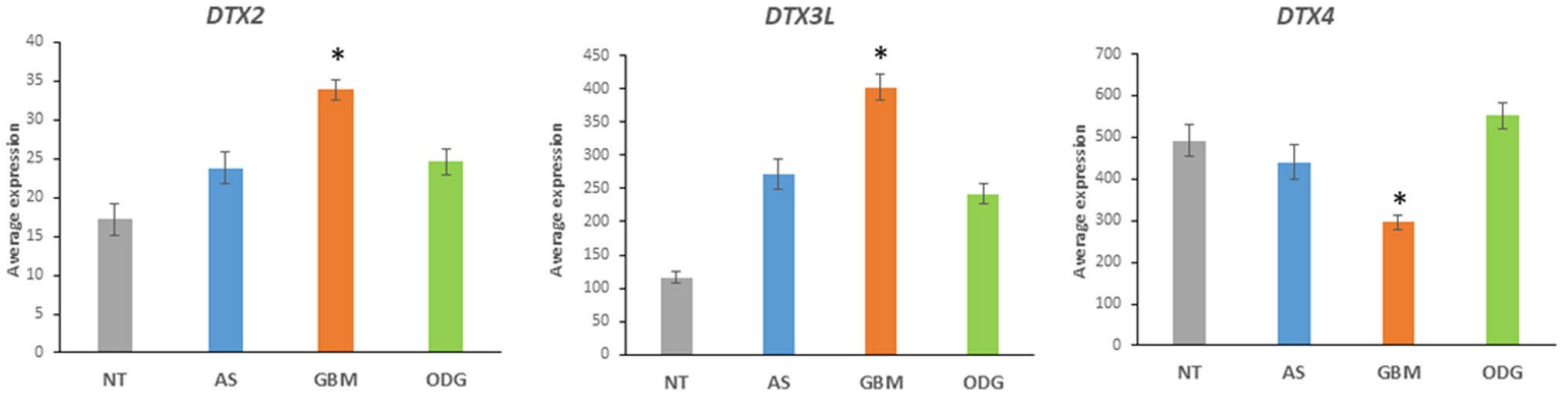
Differential expression of Deltex genes in gliomas. (*DTX2*, *F = *17.10, *p* = 9.15 × 10^−10^; *DTX3L*, *F = *33.12, *p* = 6.03 × 10^−17^; *DTX4*, *F = *22.34, *p* = 2.90 × 10^−12^). *By *t* test, expression of *DTX2* and *DTX3L* are elevated in GBM, while expression of *DTX4* is depressed compared to other groups

Expression of several genes encoding ubiquitin E3 ligases associated with the Notch pathway distinguished GBM from AS and ODG in the Sun dataset (Fig. 3). The Deltex genes, *DTX1* (aka *RNF140*), *DTX2* (aka *RNF58*), *DTX3* (aka *RNF154*), *DTX3L* (aka *RNF143*), and *DTX4* (aka *RNF155*), belong to the RING family of E3 ligases and affect the Notch signal cascade (Takeyama et al. 2003). The expression of *DTX2* and *DTX3L* was increased in GBM in the Sun dataset (*DTX2*, *F = *17.10, *p* = 9.15 × 10^*−*10^; *DTX3L*, *F = *33.12, *p* = 6.03 × 10^*−*17^), relative to AS, ODG, and the NT groups, while the expression of DTX4 was relatively decreased in GBM when compared to the other groups (*F* = 22.34, *p* = 2.90 × 10^*−*12^) (Fig. 4). Steinbuck and Winandy (Steinbuck and Winandy 2018) provide a model in which processing of the Notch receptor requires endocytosis and cleavage of the receptor. In this model, Deltex proteins contribute to the regulation of Notch receptor endocytosis and regulate the release of the intracellular domain (NICD) of the Notch receptor (Steinbuck and Winandy 2018; Fuwa et al. 2006; Schnute et al. 2018).

**Fig. 5:**
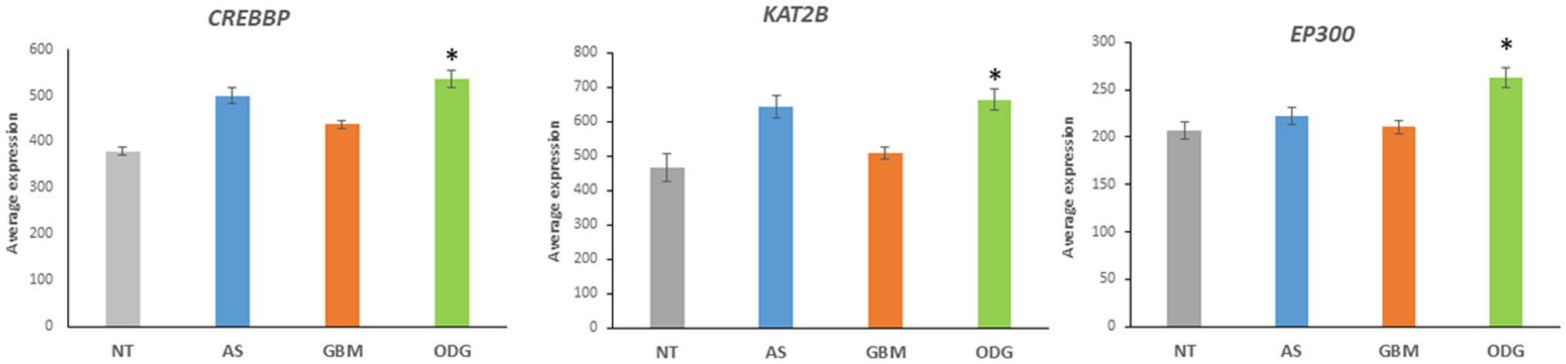
Histone acetyltransferases with E3 ligase activity are differentially expressed. (*CREBBP*, *F = *18.93, *p* = 1.18 × 10^−10^; *KAT2B*, *F = *11.21, *p* = 9.33 × 10^−7^; *EP300*, *F = *8.27, *p* = 3.58 × 10^−5^). *By *t* test, all three genes were higher expressed in ODG compared to GBM (*CREBBP*, *t = *5.36, *p* < 0.0001; *KAT2B*, *t = *4.67, *p* = 0.0001; *EP300*, *t = *4.35, *p* < 0.0001, and higher than in NT (*CREBBP*, *t = *5.69, *p* < 0.0001; *KAT2B*, *t = *3.68, *p* < 001; *EP300*, *t = *3.40, *p < *0.01), but not significantly different from AS

**Fig. 6:**
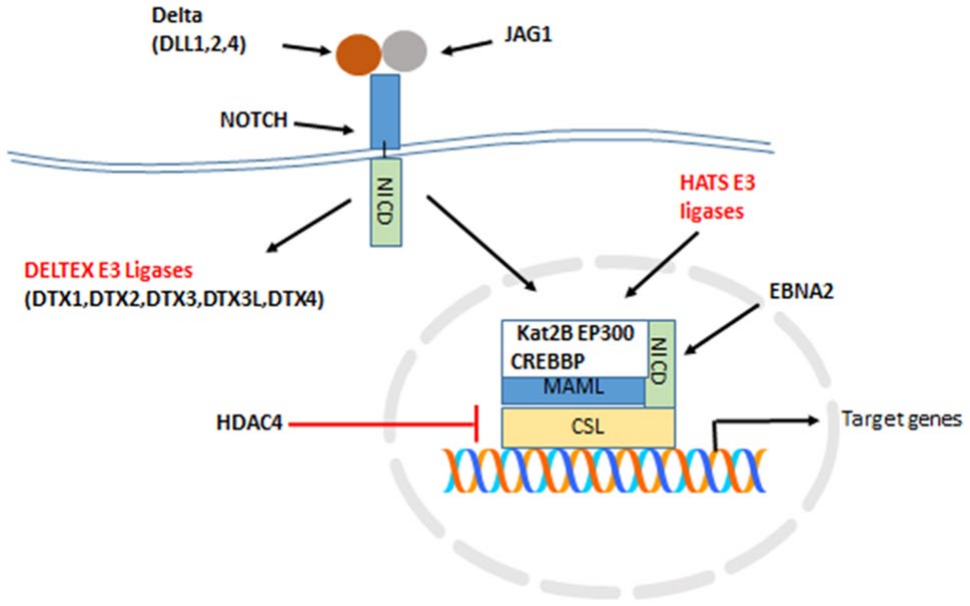
Notch signaling pathway and E3 ligases. NICD – Notch intracellular domain; HATS – Histone acetyltransferase with E3 ubiquitin ligase domain (*KAT2B*, *EP300*, *CREBBP*); EBNA2 – Epstein-Barr virus nuclear antigen

The expression of three genes in the Notch pathway, *KAT2B*, *EP300*, and *CREBBP*, which encode histone acetyltransferases with E3 ubiquitin ligase domains, was also significantly different among the three gliomas in the Sun dataset (Fig. 5). Expression of *CREBBP*, *KAT2B*, and *EP300* was significantly elevated in ODG compared to GBM (*CREBBP*, *t* = 5.36, *p* < 0.0001; *KAT2B*, *t* = 4.67, *p* < 0.0001; *EP300*, *t* = 4.31, *p* < 0.0001) and non-tumor (NT) brain tissue (*CREBBP*, *t* = 5.68, *p* < 0.0001; *KAT2B*, *t* = 3.66, *p* < 0.0005; *EP300*, *t* = 3.37, *p* < 0.01) (Fig. 5). These genes encode proteins of the canonical Notch pathway (Lee et al. 2015) and modulate the activation of the NICD (Notch intracellular domain) transcription complex together with the transcriptional regulator CSL (CBF-1, Suppressor of Hairless, Lag-2) (Fig. 6) (Maier 2006; Kopan and Ilagan 2009). The Epstein-Barr virus nuclear antigen (EBNA2) has been reported to be partially exchangeable with NICD (Fig. 6) (Zimber-Strobl and Strobl 2001; Zimber-Strobl et al. 1994). Transcriptional targets of the Notch pathway include *HES1*, *HES5*, *HES6*, *HES7*, *HEY1*, *HEY2*, *CD25*, *CCND1*, *CDKN1A*, *GATA3*, *DTX1*, and *NOTCH3* (Borggrefe and Oswald 2009; Natsuizaka et al. 2017).

*In summary*, the opposite regulation of ubiquitin E3 ligase gene clusters (Fig. 3) in GBM and OGD suggests different underlying gene regulatory mechanisms that promote distinct E3 ligase biology in GBM and ODG. Genes encoding proteins with an E3 ligase domain were involved in the dysregulation of the Notch signaling pathway in GBM. Several of these gene products are known to interact with the intracellular domain of the Notch receptor (NICD) to form a transcription complex that regulates the expression of Notch target genes (Fig. 6).

### Gene Expression for E3 Ligase Adaptors: Upregulation of Cytokine Suppressor Genes and HIPPO Pathway Genes

**Fig. 7:**
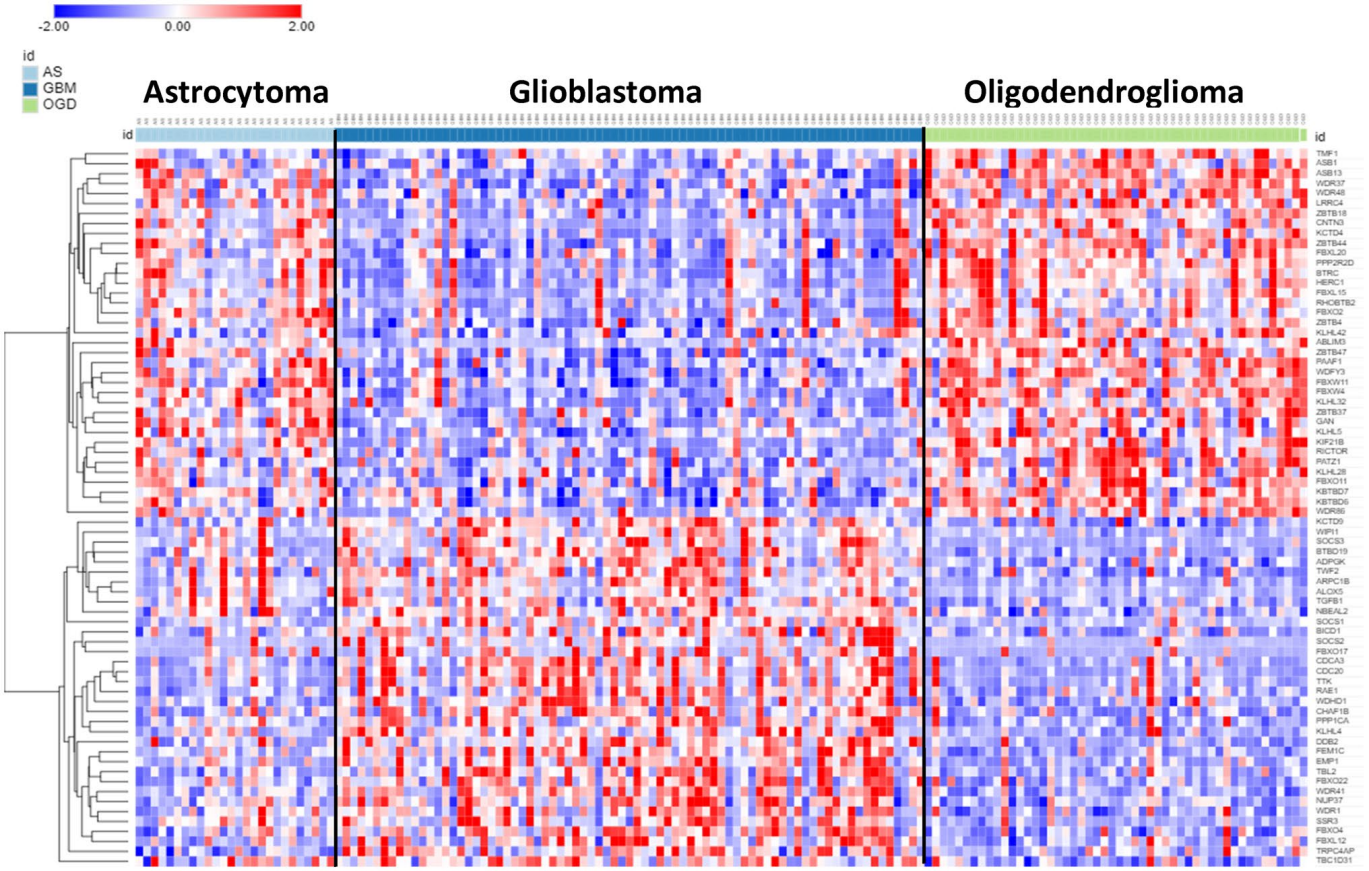
Heatmap showing relative expression of genes encoding ubiquitin E3 ligase adaptors in the Sun Glioma dataset (72 genes differentially expressed at *p < *1.0 × 10^−5^). Relative higher expression of the 35 genes (red in GBM column) and lower expression of 37 genes (blue in GBM column) were associated with lower survival in Kaplan–Meier curves derived from the French glioma dataset

Hierarchical clustering of genes encoding for E3 ligase adaptors showed opposite expression patterns in GBM versus ODG, with AS showing an intermediate expression pattern (Fig. 7). The results of the cluster analysis, depicted in the heatmap (Fig. 7), show differential gene expression (*p* < 1.0 × 10^*−*5^) of 72 genes encoding E3 ligase adaptors in AS, GBM and ODG obtained from the Sun dataset. The two E3 ligase adaptor genes relatively most significantly different (by ANOVA), *ASB13* and *KIF21B*, were expressed lower in GBM.

Two major clusters were identified with gene expression either higher (35 genes) or lower (37 genes) in GBM relative to ODG (Fig. 7). This pattern of expression is associated with significantly shorter survival in the GBM subtype. Of the 35 E3 ligase adaptor genes identified as having high expression in GBM vs ODG, 34 are associated with significantly shorter survival in the French dataset. Of the 37 genes with lower expression in GBM vs ODG, 36 are associated with significantly shorter survival in the French dataset.

The heatmap data suggest that co-regulation of transcription of a large group of genes encoding for E3 ligase adaptors may occur. Of the 72 differentially expressed E3 Cullin Ring ligase (CRL) adaptors in the Sun dataset (at *p* < 1.0 × 10^*−*5^), 23 were of the E3 adaptor/Cullin Ring DCX/DWD subfamily.

**Fig. 8:**
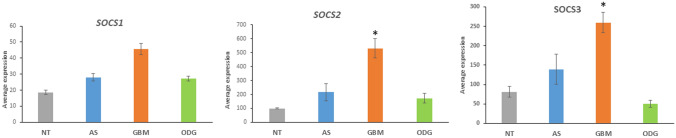
Overexpression of *SOCS* genes in GBM. *SOCS1* (*F = *13.763, *p* = 4.36 × 10^−8^), *SOCS2* (*F = *9.949, *p* = 4.40 × 10^−6^), *SOCS3* (16.728, *p* = 1.40 × 10^−9^). *By *t* test, expression of the *SOCS* genes was significantly greater in GBM than in ODG (*p < *0.0001), AS (*p < *0.05) and NT (*p < *0.01)

**Fig. 9:**
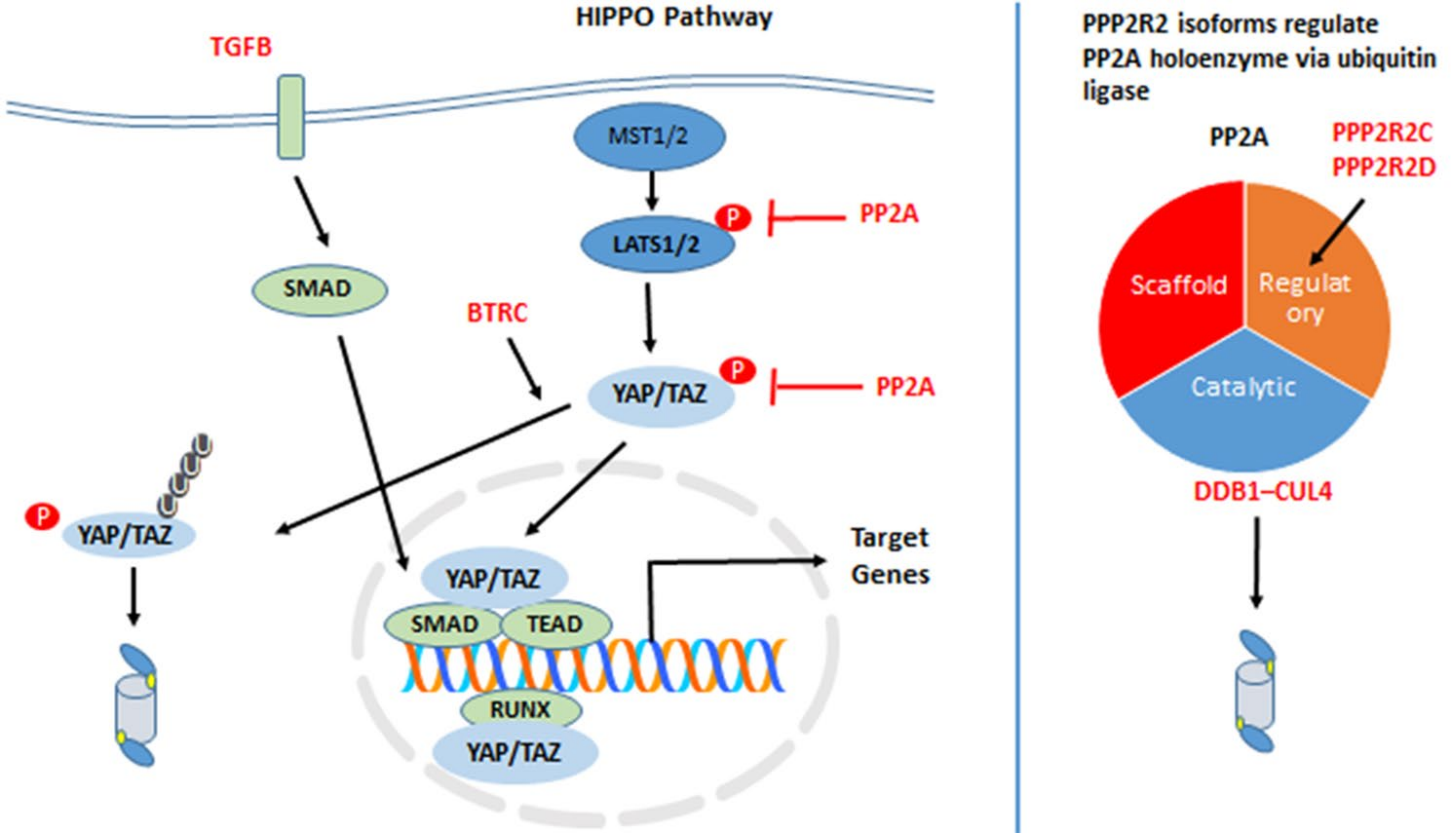
Components of the Hippo signaling pathway. Phosphorylation, ubiquitination, and TGFB/SMAD signaling converge on transcriptional coactivator YAP/TAZ and regulate YAP/TAZ-induced target gene expression. The holoenzyme protein phosphatase 2A (PP2A) contains regulatory isoforms encoded by the genes PPP2R2A, PPP2R2B, PPP2R2C, and PPP2R2D, which function as ubiquitin ligase adaptors. Dephosphorylation enables YAP/TAZ to enter the nucleus, while phosphorylation facilitates YAP/TAZ ubiquitination and degradation by the proteasome. YAP/TAZ is a coactivator for transcription factors SMAD and TEAD (Guo and Teng 2015)

The main KEGG pathways over-represented in the E3 adaptor data were the Type II diabetes pathway (*SOCS1*, *SOCS2*, *SOCS3*, suppressor of cytokine signaling genes) (Fig. 8) and the Hippo signaling pathway (Fig. 9, Table 2). Expression of the SOCS genes were relatively elevated in the GBM group (Fig. 8). Kaplan–Meier survival curves created from the French dataset show that in this dataset high expression of these genes is associated with shorter survival (*SOCS1*, *p* = 1.4 × 10^*−*13^; *SOCS2*, *p* = 6.0 × 10^*−*8^; *SOCS3*, *p* = 3.9 × 10^*−*14^).

**Table 2:** Overexpressed KEGG pathways associated with differential expression of ubiquitin ligase adaptors in the Sun dataset

KEGG Path over-representation^a^	*p* for pathway	Genes
Ubiquitin mediated proteolysis	5.8 × 10^−31^	*BTRC, CDC20, DDB2, FBXO2, FBXO4, BXW11, HERC1, RHOBTB2, SOCS1, SOCS3*
Type II diabetes mellitus	4.2 × 10^*−*8^	*SOCS1, SOCS2, SOCS3*
Hippo signaling	6.0 × 10^*−*7^	*BTRC, FBXW11, PPP1CA, PPP2R2D, TGFB1*
Oocyte meiosis	5.0 × 10^*−*6^	*BTRC, CDC20, FBXW11, PPP1CA*

^a^Using gene expression (log2) of Fig. 7 (AS, GBM, and ODG groups)

SOCS proteins are involved in regulation of several pathways including regulation of insulin signaling, growth hormone receptors signaling, and innate immunity (Huang et al. 2020). Furthermore, the SOCS proteins play a role in modulating the activity of phosphatidylinositol 3-kinase (PI-3 K) activity (Shepherd 2005). Dysregulated control of PI-3 K signaling occurs in a variety of cancers and PI-3 K inhibitors have been developed as therapeutic molecules with anticancer activity (Alzahrani 2019; Zhao et al. 2017). GO analysis showed that regulation of PI-3 K was a major biological process related to SOCS expression in the Sun dataset.

**Fig. 10:**
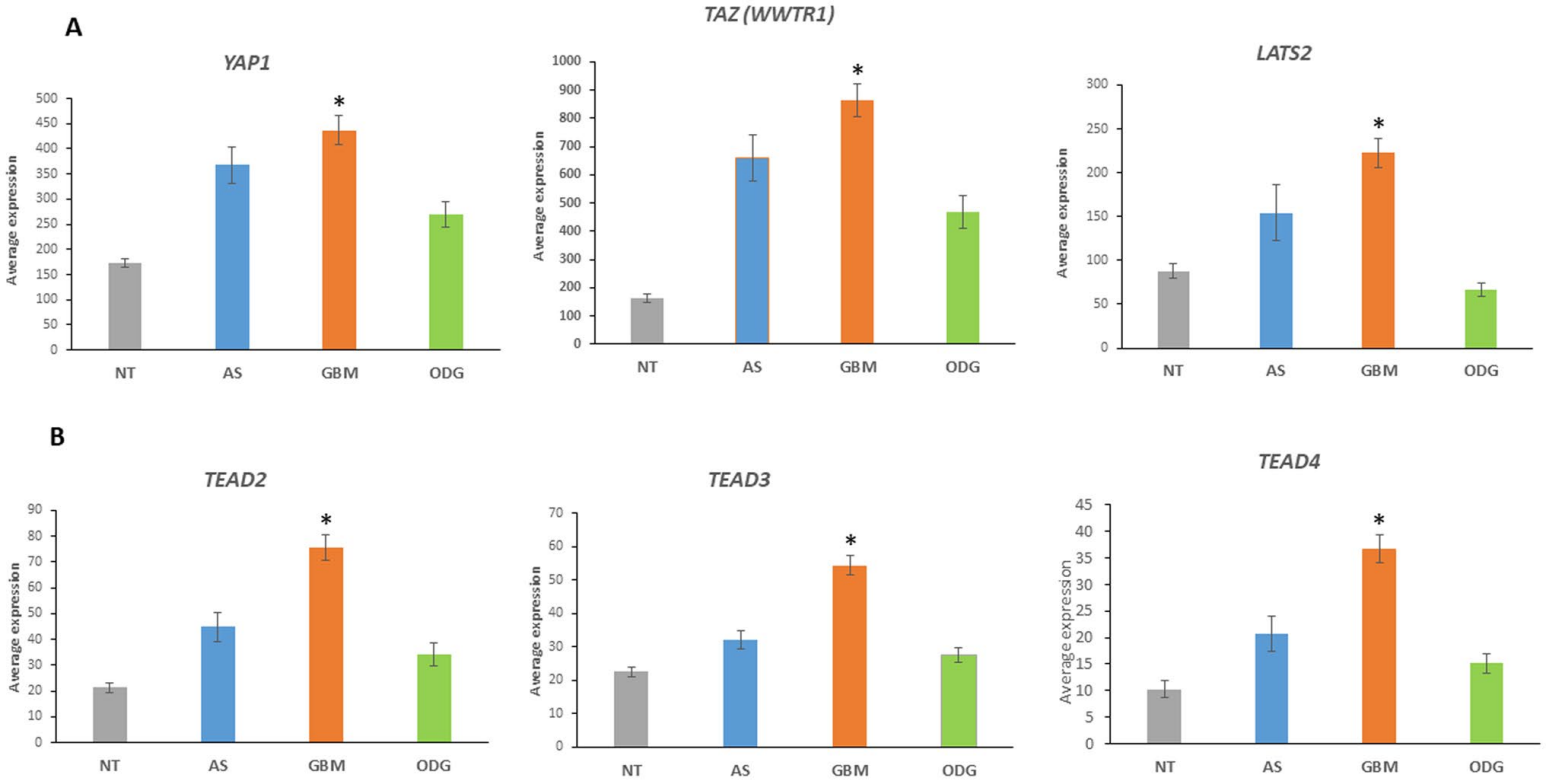
Hippo pathway genes in glioma and non-tumor (NT) tissue. **A** Increased expression of *YAP1, TAZ,* and *LATS* in GBM, compared to the ODG or NT group: *YAP1* (*F = *12.71, *p* = 1.52 × 10^−7^); *WWTR1* (*TAZ*) (*F = *19.46, *p* = 6.53 × 10^−11^); *LATS2* (*F = *20.07, *p* = 3.34 × 10^−11^); *By *t* test, GBM expression of *YAP1*, *TAZ*, and *LATS2* was significantly higher than that of NT and ODG at *p < *0.0001. Expression of these three genes in the AS group was not significantly different from GBM. **B** Upregulation of TEAD transcription in GBM. The TEAD genes encode transcription factors in the Hippo signaling pathway: *TEAD2* (*F = *21.38, *p* = 8.04 × 10^−12^); *TEAD3* (*F = *28.64, *p* = 4.53 × 10^−15^); *TEAD4* (*F = *20.56, *p* = 1.97 × 10.^−11^). The role of TEAD proteins as oncoproteins, and potential therapeutic targets, has been reviewed by Pobbati and Hong (Pobbati and Hong 2020). *By *t* test, GBM expression of *TEAD2*, *TEAD3*, and *TEAD4* was significantly higher than that of NT and ODG at *p < *0.0001, and higher than AS at *p < *0.01

The major components of the Hippo pathway are shown in Fig. 9. Genes coding for the kinase cascade of the Hippo pathway are dysregulated in several cancers (Wang et al. 2018). Six ubiquitin E3 ligase adaptor genes associated with the Hippo signaling pathway were differentially expressed in the Sun dataset. These included: *BRTC* (aka *BTrCP1*, aka *FBXW1*) *F* = 23.73, *p* = 1.11 × 10^*−*9^; *BTRC2* (aka *FBXW11*) *F* = 19.56, *p* = 2.83 × 10^*−*8^, three PPP genes encoding subunits of protein phosphatases with ubiquitin ligase E3 adaptor domains (*PPP1CA* *F* = 13.85, *p* = 3.03 × 10^*−*6^; *PPP2R2C*, *F* = 6.65, *p* = 1.71 × 10^*−*3^; *PPP2R2D*, *F* = 21.89, *p* = 4.57 × 10^*−*9^, and *TGFB1*, *F* = 15.08, *p* = 1.08 × 10^*−*6^. The statistical differences, as indicated by the F values, were also highly significant when the Sun Mixed Glioma dataset with the additional non-tumor group was analyzed (Fig. 10). These data led us to determine whether differential gene transcription of components other than E3 adaptors genes occurred in the Hippo signaling pathway.

In addition to the above ubiquitin ligase adaptors, the following genes-encoding components of the Hippo pathway were expressed significantly higher (*p* < 0.0001) in GBM than ODG and the NT group, hence, contributing to GBM signature: *STK3* (*MST2*), *YAP1*, *TAZ* (*WWTR1*), *LATS2*, *MOB1A*, *TEAD2*, *TEAD3*, and *TEAD4* (Fig. 10). The altered expression of. Hippo pathway members *YAP/TAZ*, *LATS2*, *TEAD2*, *TEAD3*, and *TEAD4* were also part of a unique signature for various other cancers (Wang et al. 2018).

**Fig. 11:**
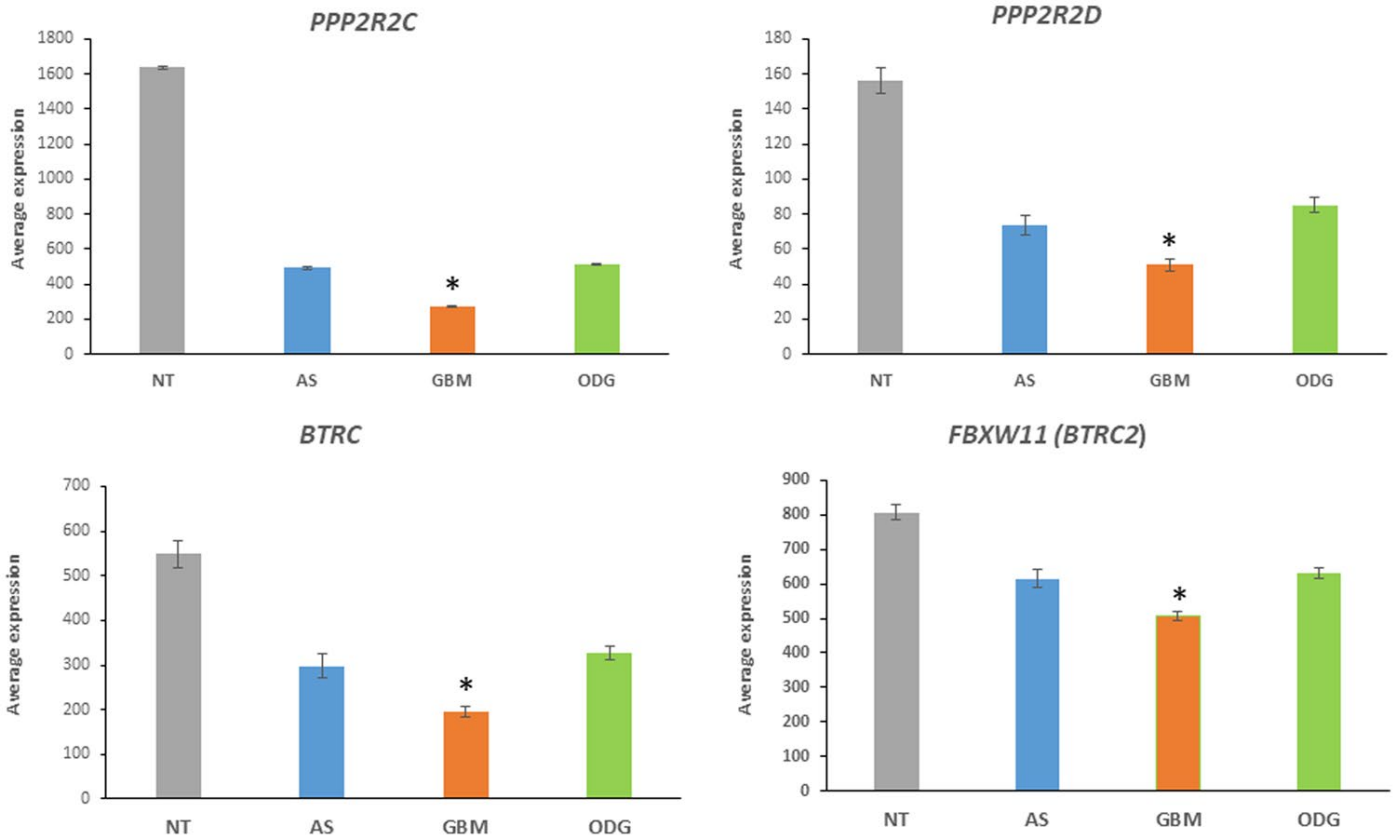
Expression of genes encoding Cullin Ring E3 ligase adaptors distinguish GBM from AS and ODG gliomas, and non-tumor tissue (NT) (*PPP2R2C*, *F = *74.68, *p* = 5.63 X 10^−31^; *PPP2R2D*, *F = *74.31, *p* = 7.15 × 10^−31^; *BTRC* *F = *57.58, *p* = 8.04 × 10^−26^; *FBXW11*, *F = *41.50, *p* = 3.14 × 10^−20^). *By *t* test, *PPP2R2C*, *PPP2R2D*, *BTRC* and *FBXW11* expression was lower in GBM than that of NT (*p < *0.0001) and lower than in AS and ODG groups (*p < *0.01)

The activity of the kinase cascade which phosphorylates and inactivates YAP/TAZ is regulated by phosphatase holoenzyme PP2A, a protein considered a regulator of tumorigenesis (Fig. 9) (Ruvolo 2016). The PP2A holoenzyme consists of three components, a structural backbone, a catalytic component and a regulatory component. According to the UUCD database, *PPP2R2C* and *PPP2R2D* encode isoforms of the B55 subfamily of PP2A regulatory components and are identified as Cullin Ring ubiquitin ligase adaptors and members of the DWD (DDB1-binding WD40 protein) subfamily. They serve as substrate recognition subunits for the DCX (DDB1-CUL4-X-box) E3 ligase complex (Angers et al. 2006; Jackson and Xiong 2009) (Fig. 9), ODG expressed higher levels of the *PPP2R2C* and *PPP2R2D* isoforms as determined in the Sun dataset (Fig. 11). These genes qualify as strong markers of a Hippo signature that distinguishes the three types of glioma. *PPP2R2C* may be a tumor suppressor gene in gliomas (Fan et al. 2013). The Sun dataset provides additional evidence that both *PPP2R2C* and *PPP2R2D* may play a role in regulation of the Hippo pathway in gliomas.

Because the genes for the transcriptional coactivators YAP/TAZ and TEAD were expressed higher in GBM than in AS, ODG, and NT samples (Fig. 10), we investigated whether this would translate into higher expression of YAP/TAZ target genes in GBM..Indeed, several YAP/TAZ target genes (*CTRG1*, *BIRC5*, *MCM3*, *MCM6*, *CDK1*, *CDC42*, *CDH2*, *SNAI1*, *SNAI2*, *VIM*) were found upregulated in GBM samples in the Sun dataset, suggesting higher activity of these Hippo transcriptional coactivators in GBM.

**Fig. 12:**
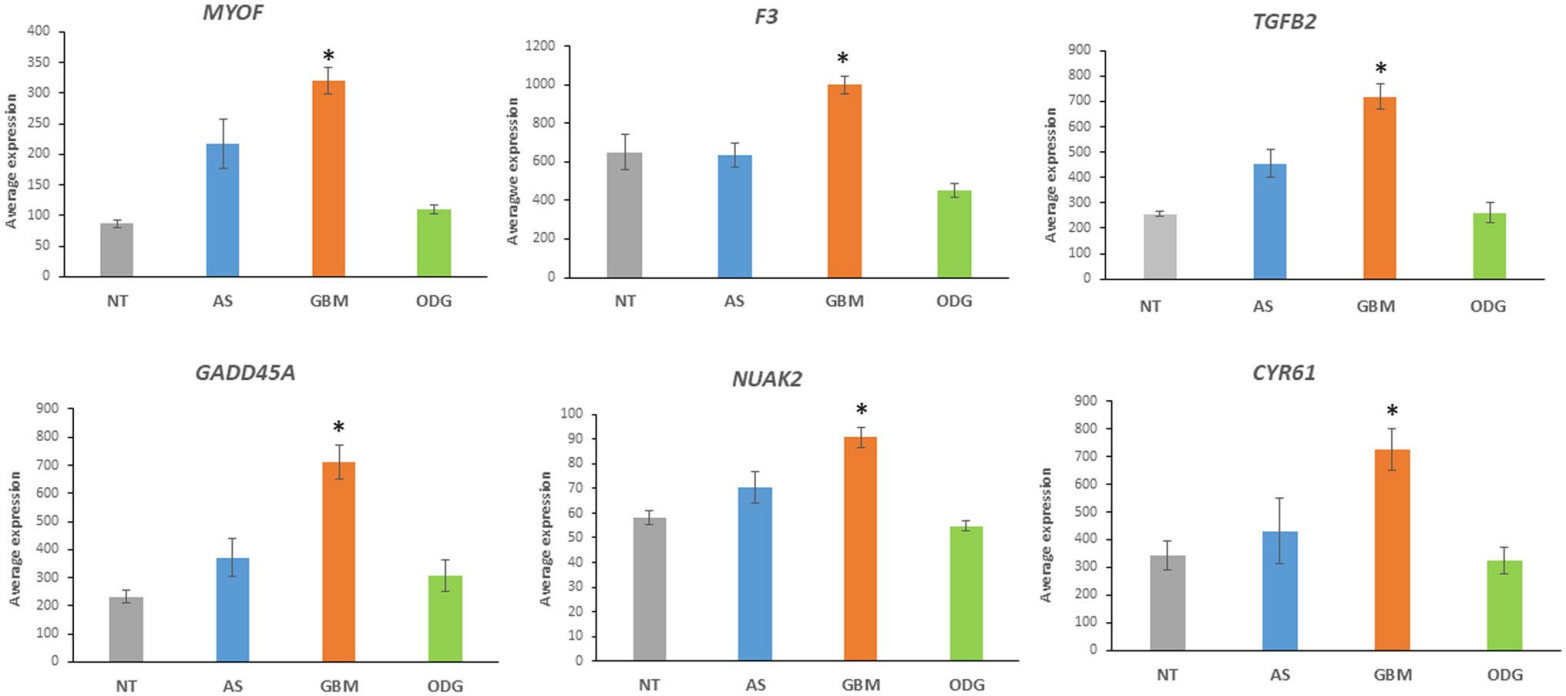
Upregulation of Hippo target genes in GBM: *MYOF*, *F = *25.24, *p* = 1.39 × 10^−13^; *F3*, *F = *15.74, *p* = 4.33 × 10^−9^; *TGFB2*, *F = *22.01, *p* = 4.11 × 10^−12^; *GADD45A*, *F = *12.80, *p* = 1.36 × 10^−7^; *NUAK2*, 19.54, *p* = 6.00 × 10^−11^; *CYR61*, *F = *7.23, *p* = 1.34 × 10^−4^. *By t test, the expression of these genes is significantly greater in the GBM (orange) group compared to the ODG group; *MYOF*, *t = *7.54; *F3*, *t = *6.19; *TGFB2*, *t = *6.53; *GADD45A*, *t = *4.56; *NUAK2*, *t = *6.89; *CYR61*, *t = *4.02, all highly significantly different, at *p < *0.001. The differences observed for these genes between the GBM and the non-tumor (NT) group were also highly significant. The differences observed for these genes between the GBM and the AS group were less significant

Wang et al. reported a cancer signature of 22 YAP/TAZ target genes (Wang et al. 2018). In the Sun dataset, 14 of these genes were significantly elevated in the GBM group compared to the ODG group and, of those, 10 genes were also significantly elevated compared to the NT group (Table 3). Figure 12 shows the most significant changes of the 22 YAP/TAZ target genes. Overall, the data showed a role for ubiquitin ligases and their adaptors with YAP/TAZ/TEAD-induced transcriptional upregulation of HIPPO pathways genes. The gene expression values suggest a sequence of events in which dephosphorylation of YAP/TAZ facilitates translocation to the nucleus, interaction with co-transcription factors, and transcription of several Hippo target genes. Figure 12 shows the upregulation of a number of Hippo target genes in GBM compared to ODG, non-tumor brain tissue (NT), and to a lesser extent also to AS.

**Table 3:** Upregulation of various Hippo target genes in GBM, compared to OGD and NT

Gene	AS *n = *26 Means ± s.e	GBM *n = *77 Means ± s.e	ODG *n = *50 Means ± s.e	NT *n = *23 Means ± s.e	F	*p*
*CYR61*	431.29 ± 119.08	725.66 ± 74.48	322.90 ± 47.75	341.70 ± 51.91	7.23	1.34 × 10^*−*4^
*CTGF*	358.85 ± 126.60	447.64 ± 52.55	217.04 ± 29.57	161.31 ± 13.97	4.73	3.73 × 10^*−*3^
*IGFBP3*	381.18 ± 102.65	1244.47 ± 147.79	309.38 ± 110.69	81.27 ± 10.8	14.58	1.66 × 10^*−*8^
*F3*	634.91 ± 90.97	998.05 ± 64.25	454.21 ± 45.47	650.93 ± 35.70	15.74	4.33 × 10^*−*9^
*NUAK2*	70.55 ± 6.57	90.83 ± 3.98	54.76 ± 2.12	58.27 ± 2.71	19.54	6.00 × 10^*−*11^
*LATS2*	154.45 ± 32.28	222.43 ± 16.36	66.12 ± 7.51	87.80 ± 7.93	20.07	3.34 × 10^*−*11^
*GADD45A*	371.71 ± 65.69	711.09 ± 62.13	306.85 ± 54.18	231.95 ± 21.34	12.80	1.36 × 10^*−*7^
TGFB2	456.54 ± 56.30	720.18 ± 50.05	261.84 ± 40.32	256.27 ± 10.12	22.01	4.11 × 10 ^−12^
PTPN14	72.71 ± 12.37	113.91 ± 7.99	55.13 ± 4.46	52.33 ± 2.58	14.94	1.10 × 10^*−*8^
MYOF	216.91 ± 40.79	320.35 ± 21.91	110.14 ± 7.49	86.89 ± 6.03	25.24	1.39 × 10^*−*13^

*In summary*, the segregation of the transcriptional datasets of genes encoding ubiquitin E3 ligase adaptors into clearly distinct high and low gene expression clusters and their opposite distribution in GBM versus ODG may reflect distinct epigenetic gene regulatory mechanisms. Intriguingly, AS had a gene expression pattern for E3 ligase adaptor intermediate to GBM and ODG. This suggest that E3 ligase adaptor genes become targets during the developmental process from lower grade AS to high-grade GBM. Notably, in GBM there was the increased expression of (i) cytokine signaling genes coding for proteins with an additional E3 ligase adaptor domain (Fig. 8) and (ii) genes related to the HIPPO pathway (Fig. 10A) that was not observed in ODG and non-tumor (NT) group. Associated with changes in the HIPPO pathway were decreased expression of phosphatase 2A regulatory genes *PPP2R2C* and *PPP2R2D* coding for proteins with an E3 adaptor domain.

### Chromosome 19 and Genes for E3 Ligases in Glioma Subtypes

Several E3 ligase adaptor genes (*FBXO17*, *TGFB1*, *SHKBP1*, *GRWD1*) differentially expressed in GBM, AS, and ODG gliomas are located on the long arm of chromosome 19 in a region associated with deletions in chromosome 19 (19q13.2 to 19q13.4) and linked to the progression of AS to secondary GBM (Nakamura et al. 2000; von Deimling et al. 1994). Emerging evidence suggests the presence of a glioma tumor suppressor gene that maps within the deletion region 19q13.3 (Smith et al. 1999; Barbashina et al. 2005; Yong et al. 1995). NUP62 is an E3 ligase gene associated with chromosome 19q13.3 (Table 4). Relative expression levels of the four E3 ligase adaptor genes *FBXO17*, *TGFB1*, *SHKBP1*, *GRWD1*, and the E3 ligase *NUP62* gene in the Sun dataset are shown in Table 4. With the exception of the E3 ligase adaptor gene *GRWD1*, the expression of the other four genes was increased in GBM relative to the ODG and NT groups (Table 4). The F-box protein FBXO17 is emerging as a new multifunctional regulator of tumorigenesis. Elevated FBXO17 expression in high-grade glioma coincided with significantly shorter overall survival of patients (Du et al. 2018). We confirmed that high gene expression of *FBXO17* was also significantly related to shorter survival (*p < *6.8 × 10^*−*21^) in the French glioma dataset.

**Table 4:** Chromosome 19 and gene expression of E3 ligases and adaptors

Gene	AS *n = *26	GBM *n = *77	ODG *n = *50	NT *n = *23	F	*p*
*FBXO17*	16.35 ± 4.39	30.69 ± 3.74*	8.66 ± 2.06	6.87 ± 0.39	10.596	1.98 × 10^*−*6^
*TGFB1*	179.53 ± 18.27	193.7 ± 10.39*	114.87 ± 6.95	78.06 ± 5.6	20.262	2.72 × 10^*−*11^
*SHKBP1*	52.27 ± 6.46	55.92 ± 2.47*	34.86 ± 2.54	31.51 ± 3.73	13.121	9.33 × 10^*−*8^
*GRWD1*	51.19 ± 2.67	64.1 ± 2.23*	51.05 ± 2.33	48.68 ± 2.57	9.189	1.13 × 10^*−*5^
*NUP62*	256.99 ± 16.65	318.3 ± 12.29*	198.27 ± 12.85	159.11 ± 8.3	25.958	6.68 × 10^*−*14^

^*^FBXO17, GBM vs AS, *p < *0.05; GBM vs ODG, *p < *0.0001; GBM vs NT, *p* < 0.001; by t test; *TGFB1, GBM vs AS, nsd; GBM vs ODG, *p* < 0.0001; GBM vs NT, *p* < 0.0001; *SHKBP1, GBM vs AS, nsd; GBM vs ODG, *p* < 0.0001, GBM vs NT, *p* < 0.01; *GRWD1, GBM vs AS, *p* < 0.01; GBM vs ODG, *p* < 0.001, GBM vs NT, *p* < 0.001; *NUP62, GBM vs AS, p < 0.01; GBM vs ODG, *p* < 0.0001, GBM vs NT, *p* < 0.0001

FBXO17 is a member of SKP1-Cullin1-F-box E3 ligase complexes that control downstream substrate stability to reduce inflammation and promote cell growth and survival. In glioma and epithelial lung cancer cells, FBXO17 targets the AKT/GSK/SNAIL signaling pathway to promote cell growth, migration, tissue invasion, and epithelial to mesenchymal transition (EMT). This may involve poly-ubiquitination and degradation of glycogen synthase kinase-3β (GSK3β) to modulate AKT/SNAIL and ERK kinase pathway activation (Suber et al. 2018; Wang et al. 2021). FBXO17 also interacts with proteins of the signalosome, a multi-protein complex that regulates ubiquitin ligases by removing Nedd8 from ubiquitin ligases (Wang et al. 2021). FBXO17 acts as a negative regulator of inflammatory immune responses by reducing type-I interferon signaling and INF-I-induced expression of Interferon-Stimulated Genes (ISGs) in immune responses to viral infections (Murira and Lamarre 2016).

The expression of the suppressor of cytokine signaling (SOCS) E3 adaptor genes, *SOCS1*, *SOCS2*, and *SOCS3* (Fig. 8) are stimulated by IFN gamma (Song and Shuai 1998) and SOCS1 protein contributes to the regulation of the INF-induced immune response to infections (Fenimore and Young 2016). The SOCs genes are dysregulated in several tumors including GBM (Zhou et al. 2007). The Sun dataset supports the view that SOCS1-3 and downstream signaling may contribute to GBM tumorigenesis (Fig. 8).

There is a role for TGFB signaling in tumorigenesis and HIPPO signaling (Liu et al. 2018; Zhu et al. 2017). TGFB1 has a role in cell-cycle regulation by inhibiting entry into S phase (Mukherjee et al. 2010) and has been shown to provide therapeutic targets in glioma (Han et al. 2015). Antisense oligonucleotides were used to target the TGFB pathway in a mouse model of GBM (Han et al. 2015). Little is known regarding any role for GRWD1 (Takafuji et al. 2017), NUP62 (Nofrini et al. 2016), and SHKBP1 in glioma tumorigenesis. The E3 ligase NUP62 interacts with the centrosome and mitotic spindle during mitosis (Hashizume et al. 2013; Borlido and D'Angelo 2014) (89, 90) and knockdown of NUP62 resulted in mitotic arrest and reduced cellular growth in several cancer cell lines.(Hashizume et al. 2013; Kinoshita et al. 2012).

### Cell-Cycle Gene Expression in Gliomas

**Fig. 13:**
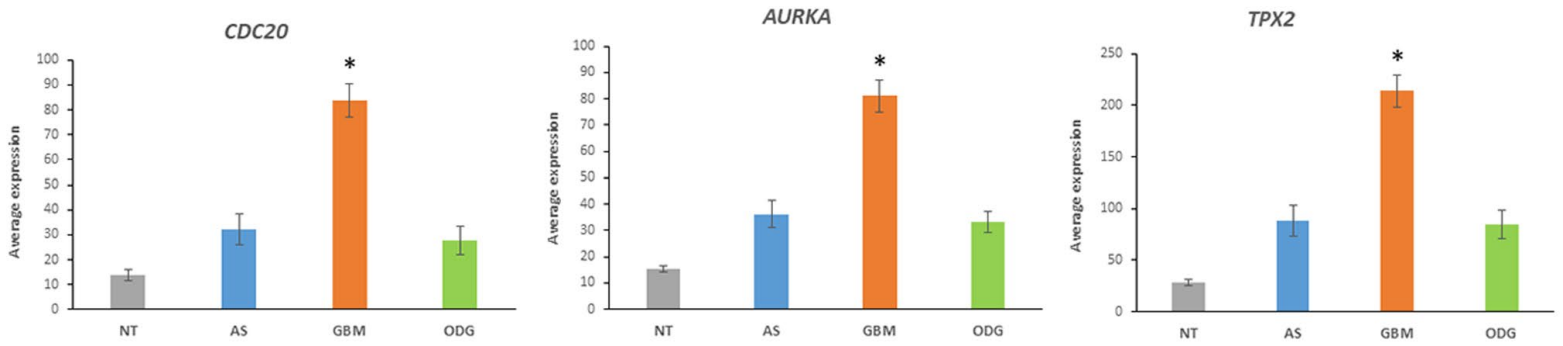
Differential expression of cell-cycle genes in Sun dataset. *CDC20* (*F = *23.21, *p* = 1.15 × 10^−12^), *AURKA* (*F = *24.76, *p* = 2.29 × 10^−13^), *TPX2* (*F = *24.73, *p* = 2.37 × 10^−13^). *By *t* test, GBM means are significantly higher than in the other groups for each gene at *p < *0.0001

Ubiquitin ligases and their adaptors have key roles in cell-cycle control (Nakayama and Nakayama 2006). High-grade tumors, including GBM, tend to escape UPS mediated mechanisms that control cell-cycle progression (Vlachostergios et al. 2012). The APC/c complex and its activator CDC20 are implicated as major regulators of the cell cycle in GBM. The APC/c complex controls mitotic exit and re-initiates gene transcription in the cell cycle (Oh et al. 2020; Vlachostergios et al. 2013). CDC20 is a key protein in control of the cell cycle by serving as an adaptor of the APC/c E3 ligase complex which mediates spindle checkpoint and protects from genomic instability (Nakayama and Nakayama 2006). The *CDC20* gene was expressed several fold higher in GBM than AS and ODG in the Sun dataset (Fig. 13). Other genes encoding subunits of three ubiquitin ligase adaptors that have important roles in the control of cell cycle are among those most differentially expressed between the three gliomas in the Sun dataset. The two most significantly differentially expressed cell-cycle genes for E3 ligases were *AURKA* and *TPX2*; the latter encodes an allosteric modulator of AURKA (Abdelbaki et al. 2020). The cell-cycle genes encoding E3 ligase adaptors with the most significantly different expression were *CDC20* (Fig. 13), *PPP2R2D* and *BTRC (*Fig. 11)*.* PPP2R2D is a regulatory component of the holoenzyme PP2A (protein phosphatase 2) described as a ‘master regulator’ of the cell cycle (Wlodarchak and Xing 2016). Expression of genes *PPP2CA* and *PPP2CB* encoding the catalytic subunits of PP2A, was not significantly different (*p > *0.05) between AS, GBM, and ODG. Decreased activity of PPP2R2D is expected to regulate the availability of substrates to the PP2A holoenzyme.

*In summary*, over-expression of the gene encoding the E3 ubiquitin ligase adaptor CDC20, an activator of the APC/C, or cyclosome, complex that targets cell-cycle proteins for degradation was noted in GBM. Also elevated was the gene encoding the serine/threonine kinase and E3 ligase AURKA, a protein that has a significant regulatory role in several mitotic events.

### Regulation of Stem Cell Differentiation and Glioma

There were 47 genes differentially expressed in the gene ontology category of “*Regulation of stem cell differentiation”* at *p < *0.01 in the Sun dataset. Table 5 shows that statistically the most significant KEGG pathway associated with the ontology category of “*Regulation of stem cell differentiation*” was the proteasome pathway (*p* = 8.6 × 10^*−*175^). Genes in this category all encoded subunits of the proteasome. Expression of the 11 genes encoding proteasome subunits depicted in Table 5 were all significantly increased (*p* < 0.001) in GBM compared to AS and ODG. This is consistent with increased transcription of genes encoding proteasome subunits and increased number of proteasomes in GBM. High proteasomal activity has been associated with enhanced cancer cell survival in selected cancers (Soave et al. 2017).

**Table 5:** Top KEGG pathways associated with ‘*Regulation of stem cell differentiation’* in Sun dataset

KEGG Pathway Over-represented^a^	*p* value (for pathway)	Genes of pathway
Proteasome	8.6 × 10^*−*175^	*PSMA1, PSMA2, PSMA3, PSMA4, PSMA5, PSMA7, PSMB10, PSMB2, PSMB3, PSMB4, PSMB8, PSMB9, PSMC2, PSMC4, PSMD8*
Hippo signaling pathway	5.4 × 10^*−*5^	*BMP7, BMPR1A, TEAD2, TGFB2, YAP1*
Notch signaling	5.8 × 10^*−*5^	*HES1, HES5, JAG1*

^a^All differentially expressed genes (log2) in the Sun dataset

The data of Table 5 suggest a link between increased proteasome activity and survival of GBM cells. *PSMB8*, *PSMB9*, and *PSMB10* encode catalytic subunits of the immunoproteasome, a modified proteasome for the processing of class I peptides of the major histocompatibility complex (MHC) and viral proteins (Hewitt 2003). The genes *PSMB8* and *PSMB9* are located in the MHC region of chromosome 6.

There were 25 genes differentially expressed in the gene ontology category of “*Regulation of stem cell proliferation*” (*p* < 0.01). The main KEGG pathway associated with the gene ontology category of “*Regulation of stem cell proliferation*” in the Sun dataset was the Hippo pathway (Table 6). The role of the Hippo signaling pathway in stem cell proliferation has been reviewed by Mo and colleagues (Mo et al. 2014). Among the differentially expressed genes contributing to this pathway was *TGFB1*, which is a gene on chromosome 19 encoding a Cullin Ring ubiquitin ligase adaptor (Table 4) and a cytokine involved in regulating immune response to cancer cells (Hargadon 2016).

**Table 6:** Top KEGG pathways associated with ‘*Regulation of stem cell proliferation*’ in Sun dataset

KEGG Pathway Over-represented^a^	*p* (for pathway)	Genes
Hippo signaling	6.5 × 10^*−*15^	*CTNNA1, NF2, SNAI2, TGFB1, YAP1*
Hedgehog signaling	2.0 × 10^*−*6^	*GLI3, SMO*
Basal cell carcinoma	8.8 × 10^*−*6^	*GLI3, SMO*
Pathways in cancer	1.7 × 10^*−*5^	*CTNNA1, GLI3, TGFB1, VEGFA*

^a^Differentially expressed genes (log2) at *p* < 0.001 (3 groups AS, GBM, and ODG)

### Differential Expression of Deubiquitinases (DUBs) in Glioma Subtypes

DUBs in GBM are emerging targets for the treatment of GBM (Jin et al. 2017). The expression of several genes encoding DUBs was depressed in GBM compared to AS and ODG in the Sun glioma dataset. In the order of statistical significance, these genes were USP46 (*F* = 36.50, *p* = 1.22 × 10^*−*13^), USP54 (*F* = 28.48, *p* = 3.28 × 10^*−*11^) ZRANB1 (*F = *25.37, *p* = 3.24 × 10^*−*10^, and OTUD7A (*F = *9.94, *p* = 8.80 × 10^*−*05^). To the best of our knowledge, these genes are currently not associated with GBM in the literature. TNFAIP3, listed in the UUCD database as having both E3 ligase and deubiqutinase activity, was also differentially expressed (*F = *11.786, *p* = 1.76 × 10^*−*5^) with significantly higher gene expression in GBM than in ODG and NT, but not AS.

### Differential Expression of Genes Encoding Proteasomal Subunits

**Fig. 14:**
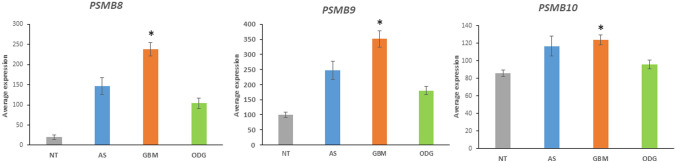
Increase in expression of *PSMB8*, *PSMB9* and *PSMB10* in GBM. *PSMB8*, *F = *26.01, *p* = 6.35 × 10^−14^; *PSMB9*, *F = *16.62, *p* = 1.59 × 10^−9^; *PSMB10*, *F = *6.90, *p* = 2.5 × 10^−4^. NT – Non-tumor group. *By *t* test, GBM is significantly elevated compared to ODG (*PSMB8*, *t = *5.71, *p < *0.0001; *PSMB9*, *t = *4.85, *p < *0.0001; *PSMB10*, *t = *3.54, *p < *0.001) and significantly elevated compared to NT (*PSMB8*, *t = *7.05, *p < *0.0001; *PSMB9*, *t = *5.04, *p < *0.0001; *PSMB10*, *t = *3.68, *p < *0.001). Expression levels of AS were of less significance or not significantly different from GBM (*PSMB8*, *t = *2.89, *p < *0.01; *PSMB9*, *t = *2.11, *p < *0.05; *PSMB10*, *t = *0.398, *p > *05)

KEGG analysis of the gene ontology (GO) category of ”Proteasome” indicated that the proteasome pathway significantly differentiated GBM, AS, and ODG in the Sun dataset. The extremely high level of significance indicated for this pathway (*p* = 3.2 × 10^*−*315^) suggested the importance of this pathway for distinguishing these three gliomas. Expression of 20 genes encoding proteasome subunits was significantly elevated in GBM compared to ODG. Elevated expression was most apparent for genes encoding catalytic subunits of the immunoproteasome, *PSMB8*, *PSMB9* (and to a lesser extent *PSMB10*), but no significant difference was observed for the genes encoding the catalytic subunits of the normal proteasome (*PSMB5*, *PSMB6*, and *PSMB7*) (Arellano-Garcia et al. 2014) (Fig. 14).

**Fig. 15:**
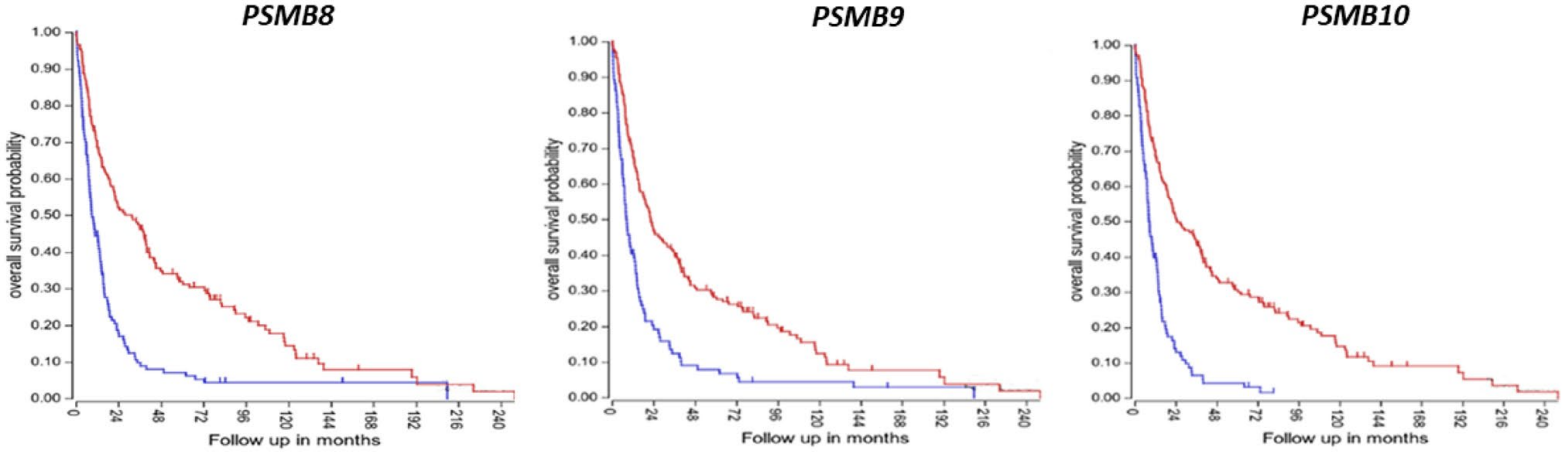
Kaplan–Meier survival curves from French dataset. Blue – high expression; Red – low expression. High expression of *PSMB8*, *PSMB9*, and *PSMB10* is associated with shorter survival. The curves were generated using the R2 genomics and visualization platform

We generated Kaplan–Meier survival curves with the French glioma dataset. High expression of *PSMB8*, *PSMB9*, and *PSMB10* was associated with shorter survival (*p* = 3.9 × 10^*−*10^, *p* = 3.5 × 10^*−*08^, *p* = 1.403 × 10^*−*13^, respectively) (Fig. 15). Considering the data showing that high expression of immunoproteasome genes is associated with shorter survival (Fig. 15) it would be useful to test immunoproteasome-specific inhibitors in GBM. Because of the association of the immunoproteasome with various diseases immunoproteasome-specific inhibitors have been developed (Ettari et al. 2018; Morozov and Karpov 2019).

Additionally, GBM showed elevated expression of the P*SME1* and *PSME2* genes encoding proteins of the 11S regulatory subunit of the immunoproteasome. Like PSMB8 and PSMB9, these immunoproteasome subunits are IFNγ target genes (Kohda et al. 1998). Masucci (2004) reviewed Epstein-Barr virus-induced (EBV) oncogenesis in relation to the UPS system and discussed the role of EBV latency proteins and the proteasome in oncogenesis (Masucci 2004). Frisan and colleagues, cited in the review by Masucci (2004), reported that the EBV latency membrane protein, LMP-1, was associated with interferon-γ-induced increase in proteasome subunits LMP7, LMP2, and MECL1 (proteins coded for by *PSMB8*, *PSMB9*, and *PSMB10*, respectively) (Masucci 2004; Frisan et al. 1998). The proteasome ubiquitin receptor *PSMD16* (aka adhesion regulating molecule 1) was among the genes encoding for proteasome subunits with elevated expression in GBM. This gene has been described as an oncogene over-expressed in several tumors and the PSMD16 gene product has recently been identified as a potential therapeutic target of multiple myeloma and ovarian cancer (Fejzo et al. 2013; Song et al. 2021).

*In summary*, the major KEGG biological pathways associated with “*Regulation of stem cell differentiation*” in the SUN dataset were the proteasome pathway, the HIPPO signaling pathway, and the Notch signaling pathway. The major KEGG pathway associated with ‘*Regulation of stem cell proliferation’* was the HIPPO signaling pathway. Overexpression of the genes for the immunoproteasome, *PSMB8*, *PSMB9*, and *PSMB10*, was noted in the GBM group compared to the OGD group and NT group. High expression of these genes was associated with shorter survival.

### Differential Expression of UPS Genes in GBM Subtypes

#### Gene Encoding Ubiquitin-Activating and Conjugating Proteins in GBM Subtypes

Expression of the gene for the ubiquitin activating enzyme UBA1 was not significantly different between classical, mesenchymal, and proneural GBM subtypes. However, GBM subtypes showed significant differences in the expression of genes encoding ubiquitin-conjugating enzymes in the TCGA dataset. Gene expression of ubiquitin conjugases *UBE2O*, *UBE2E3*, *UBE2I*, *UBE2N*, *UBE2NL*, *UBE2C*, and *UBE2S* was elevated in the proneural GBM subtype compared to classical and mesenchymal GBM subtypes (Table 7).

**Table 7:** Differential expression of genes encoding ubiquitin conjugases in TCGA Dataset

TCGA	Classical (Means ± se)	Mesenchymal (Means ± se)	Proneural (Means ± se)	F	P
	*N* = 17	*N* = 27	*N* = 24		
*UBE2O*	28.90 ± 1.73	27.44 ± 1.42	41.99 ± 2.46	17.84	6.65 × 10^*−*7^
*UBE2E3*	665.19 ± 33.30	560.68 ± 25.09	801.15 ± 43.59	13.34	1.40 × 10^*−*5^
*UBE2I*	429.11 ± 23.49	467.84 ± 18.30	636.05 ± 41.99	12.84	2.00 × 10^*−*5^
*UBE2N*	855.45 ± 31.74	777.57 ± 26.87	992.43 ± 37.08	12.54	2.50 × 10^*−*5^
*UBE2NL*	70.11 ± 3.96	58.49 ± 3.11	86.25 ± 5.07	12.63	2.33 × 10^*−*5^
*UBE2C*	152.29 ± 14.25	132.69 ± 15.56	310.10 ± 42.37	12.08	3.47 × 10^*−*5^
*UBE2S*	225.11 ± 24.09	126.91 ± 14.27	322.08 ± 49.24	9.61	2.21 × 10^*−*4^

*UBE2O* encodes a protein that is an E2/E3 hybrid enzyme and has numerous substrates. UBE2O is involved in cancer and disease onset and Ullah et al. 2018 reported that high expression of *UBE20* was associated with low survival in several cancer types (Ullah et al. 2019). *UBE2O* gene expression, along with increased expression of genes for several other ubiquitin conjugases, was relatively higher in the proneural than the other two GBM subtypes. The ubiquitin conjugases UBE2C and UBE2S are involved in cell-cycle regulation (Presta et al. 2020; Zhang et al. 2021) and have been proposed as therapeutic targets for several cancers. The current data suggest that selected conjugases are potentially lucrative targets for the treatment of proneural GBM.

*In summary*, genes for several E2 conjugases, including *UBE2C* and *UBE2S* involved in the regulation of the cell cycle, were differentially expressed in the three GBM subtypes and are potential therapeutic targets.

### Ubiquitin E3 Ligase Genes and GBM Subtypes

**Fig. 16:**
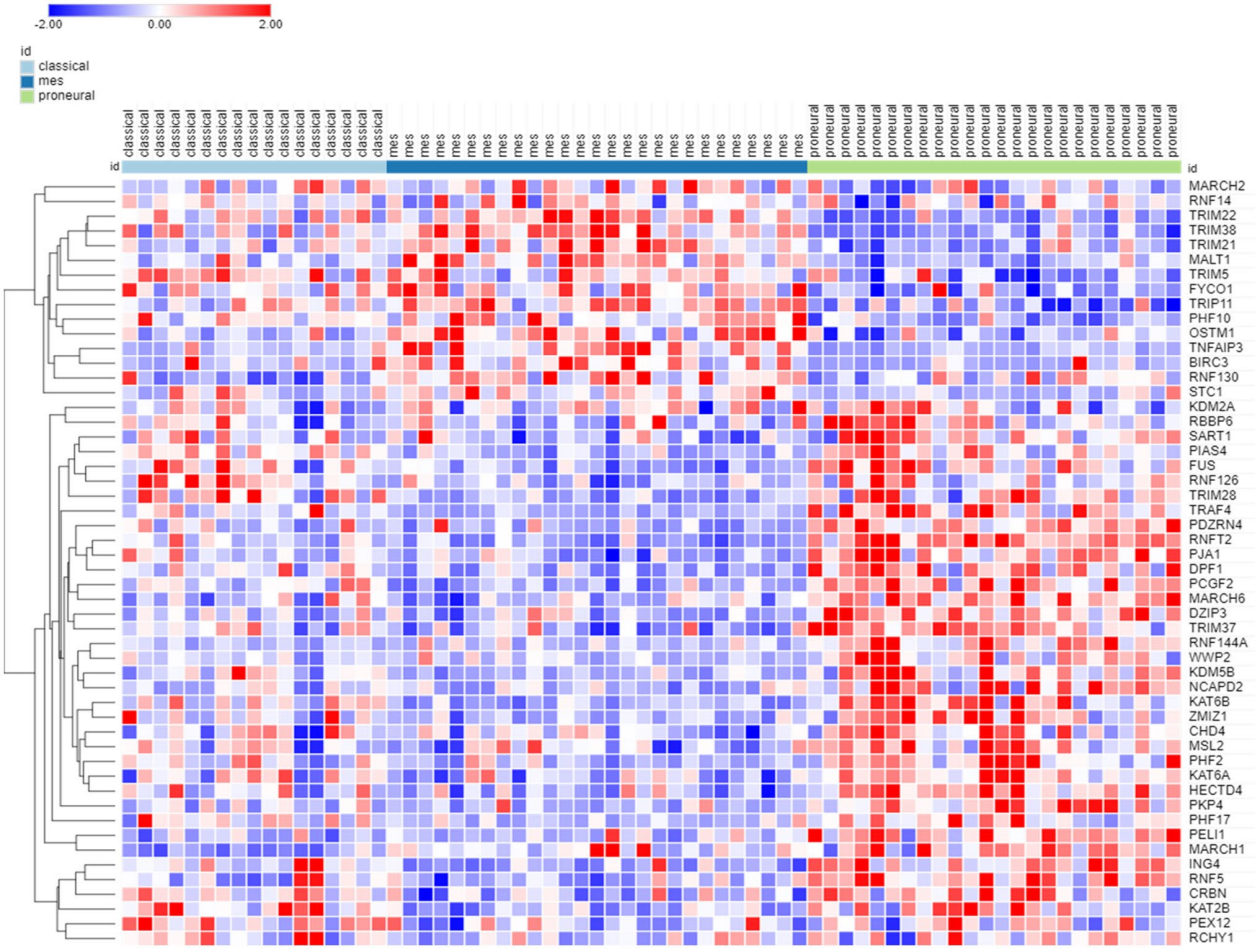
Expression of E3 ubiquitin ligases in GBM subtypes (52 genes with cutoff of *p < *0.0001). Shorter survival times was associated with 13/15 E3 ubiquitin ligase genes with higher expression in the mesenchymal subtype (red in mesenchymal column) and 25/37 genes with lower expression in the mesenchymal subtype (blue in mesenchymal column)

The results of the hierarchical cluster analysis, depicted in the heatmap (Fig. 16), show differential gene expression (with a cutoff of *p* < 1.0 × 10^*−*4^) of 52 genes encoding E3 ligases in GBM subtypes (classical, mesenchymal, proneural) in the TCGA GBM dataset. E3 ligases segregated into two major clusters with gene expression either higher or lower in the mesenchymal subtype relative to the proneural GBM group (Fig. 16). No cluster of genes was detected in the AS group. Since the TCGA GBM dataset included survival data, we were able to relate gene expression to survival. High expression of 13/15 genes encoding E3 ubiquitin ligases in the mesenchymal subtype (red in mesenchymal column) was associated with significantly shorter survival. The most significant of these genes was BIRC3, which codes for a protein which contributes to regulation of NF-kB (Yamato et al. 2015).

The main KEGG category associated with differential expression of E3 ligases in the TCGA glioblastoma dataset is the NF-kB signaling pathway (Table 8). This pathway is involved in regulation of many aspects of immune responses (Hayden et al. 2006; Zhang et al. 2017). Several E3 ubiquitin ligase genes differentially expressed in GBM subtypes are involved in the regulation of the NF-kB signaling pathway. The *BIRC3* gene product is not only a regulator of NF-kB, but also an inhibitor of apoptosis (Frazzi 2021) which is reported to facilitate progression of low-grade gliomas to higher grade gliomas (Gressot et al. 2017). *MALT1* also encodes a protein involved in NF-kB signaling (Gressot et al. 2017) and is a potential therapeutic target in GBM (Liu et al. 2020b). Interestingly, *BIRC3-MALT1* translocations have been reported in lymphomas (Schreuder et al. 2017). *PIAS4* encodes an inhibitor of NF-kB signaling (Wang et al. 2017) and TNFAIP3, listed as both an E3 ligase and a deubiquitinase in the UUCD database, regulates NF-kB activation and apoptosis (Das et al. 2018).

**Table 8:** Over-represented KEGG pathways associated with differential expression of ubiquitin ligases in TCGA dataset

KEGG pathway over-represented*	*p* value	Genes
NF-kB signaling	9.4 × 10^*−*12^	*BIRC3, MALT1, PIAS4, TNFAIP3*
Ubiquitin mediated proteolysis	6.7 × 10^*−*8^	*BIRC3, PIAS4,TRIM37,WWP2*

^*^p < 0.0001 log2 transform for KEGG analysis

*TRIM37* encodes a protein that inhibits inflammatory responses induced by virus infection (Zhao et al. 2021). *WWP2* has been described as an oncogene whose expression is dysregulated in various tumors (Zhang et al. 2019). It has been reported to interact with one of the latent proteins of the EB virus (Ikeda et al. 2000).

*In summary*, two major clusters of E3 ligases genes distinguish the mesenchymal subtype from the proneural subtype (Fig. 16). Overexpression of genes in one cluster and under-expression in a second cluster was associated with shorter survival in the mesenchymal GBM subtype. KEGG pathway analysis indicated that the NF-kB pathway, which is involved in regulation of the immune response, was significantly over-represented among these E3 ligase genes.

**Fig. 17:**
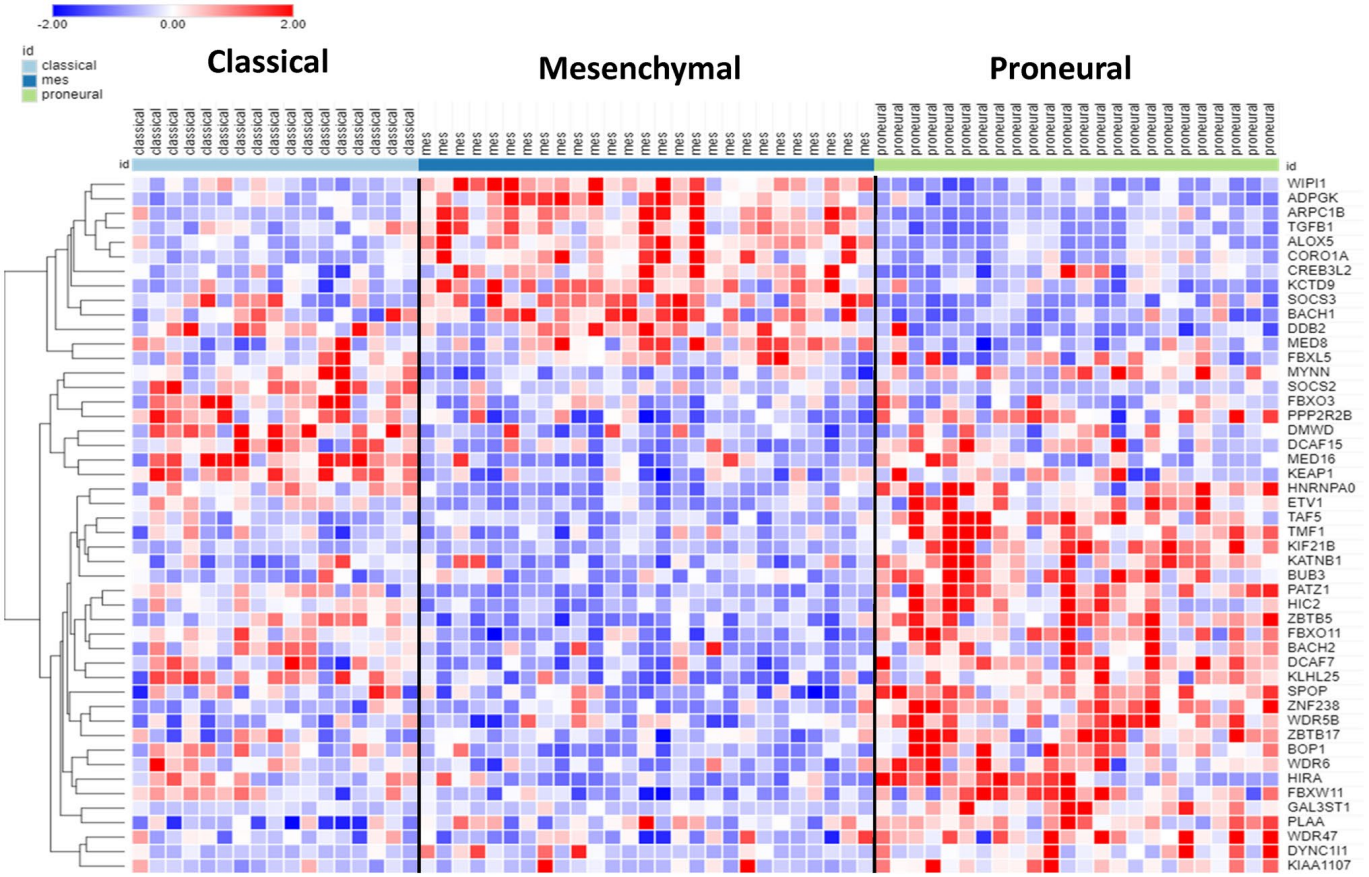
Expression of E3 ligase adaptor genes in GBM subtypes. Forty eight genes were differentially expressed at *p < *0.0001. Higher expression of 12/12 genes in the mesenchymal GBM subtype (red cluster in mesenchymal column) compared to proneural GBM and lower expression of 22/30 genes in the mesenchymal subtype (blue cluster in mesenchymal column) compared to proneural GBM subtype was associated with significantly shorter survival

The results of the hierarchical cluster analysis, depicted in the heatmap (Fig. 17), illustrate differential gene expression (with an ANOVA cutoff of *p < *1.0 × 10^*−*4^) of 48 genes encoding E3 ligase adaptors in classical, mesenchymal, and proneural GBM subtypes. Gene expression for E3 ligase adaptors segregated into two major clusters with gene expression either higher or lower in the mesenchymal GBM subtype relative to the proneural GBM subtype (Fig. 17). TCGA survival data showed that higher expression of 12/12 genes in the mesenchymal subtype (red cluster in mesenchymal column) compared to proneural GBM and lower expression of 22/30 genes in the mesenchymal GBM subtype (blue cluster in mesenchymal column) compared to proneural GBM was associated with significantly shorter survival. These data are consistent with reports that patients with mesenchymal GBM subtype have a worse prognosis than patients with other GBM subtypes.

The main KEGG pathways associated with expression of these E3 adaptor genes were *Ubiquitin mediated proteolysis*, *VP regulated water metabolism*, *Type II diabetes*, *Cell cycle*, and *Hippo signaling* (Table 9). By removing one group at a time from the analysis we identified the Hippo signaling pathway as the most likely pathway associated with the mesenchymal GBM subtype. *FBXW11* and *PPP2R2B* expression were both significantly reduced in the MES group (*F = *15.46, p = 3.22e^−06^), *F = *13.07, p = 1.70e^−05^), while *TGFB1* expression was significantly elevated in the mesenchymal GBM group (*F = *19.22, p = 2.77 × 10^*−*7^). Decreased expression of the SOCS genes (*SOCS1*, *SOCS2*, *SOCS3*) and elevated expression of the cell-cycle genes *BUB3* and *ZBTB17* was associated with the proneural GBM subtype. *CREB3L2* encodes a transcriptional factor reportedly upregulated in malignant glioma (Sampieri et al. 2019). Elevated *CREB3L2* expression was associated with the mesenchymal subtype in the TCGA GBM dataset.

**Table 9:** Over-represented KEGG pathways associated with differential expression of ubiquitin ligase adaptors in GBM subtypes (TCGA dataset)

KEGG Pathway over-represented	p value for pathway	Genes
Ubiquitin mediated proteolysis	1.3 × 10^*−*5^	*DDB2*, *FBXW11*, *KEAP1*, *SOCS3*
VP regulated water metabolism	5.5 × 10^*−*4^	*CREB3L2*,*DYNC1I1*
Type II diabetes mellitus	1.9 × 10^*−*3^	*SOCS2*, *SOCS3*
Cell cycle	2.8 × 10^*−*3^	*BUB3*, *TGFB1*, *ZBTB17*
Hippo signaling	6.4 × 10^*−*3^	*FBXW11*, *PPP2R2B*,*TGFB1*

*In summary*, the ubiquitin E3 ligase adaptor heatmap data (Fig. 17) distinguished primarily the mesenchymal and proneural subtypes. Biological pathways over-represented among these genes include pathways involving the cytokine suppressor signaling genes, cell-cycle genes, and genes of the HIPPO signaling pathway.

### The UPS and Regulation of Stem Cell Differentiation and Proliferation in GBM

Among the three GBM subtypes, 36 genes were significantly different (*p < *0.01) in the gene ontology category of “*Regulation of stem cell differentiation*” in the TCGA dataset. The KEGG pathways statistically over-represented in this group of genes were the “*Proteasome*” and “*Notch signaling pathways*,” These data provide evidence that expression of proteasome genes could distinguish classic, mesenchymal, and proneural GBM subtypes (Table 10).

**Table 10:** Top KEGG pathways associated with “*Regulation of stem cell differentiation*” in TCGA dataset

KEGG Pathway Over-represented	*p* (for pathway)	Genes
Proteasome	2.4 × 10^−92^	*PSMB10,, PSMB7, PSMB8, PSMB9, PSMC2, PSMD1, PSMD3, PSME1, PSME2, PSMF1*
Notch signaling	1.5 × 10^−07^	*HES1, JAG1, NOTCH1*

**Fig. 18:**
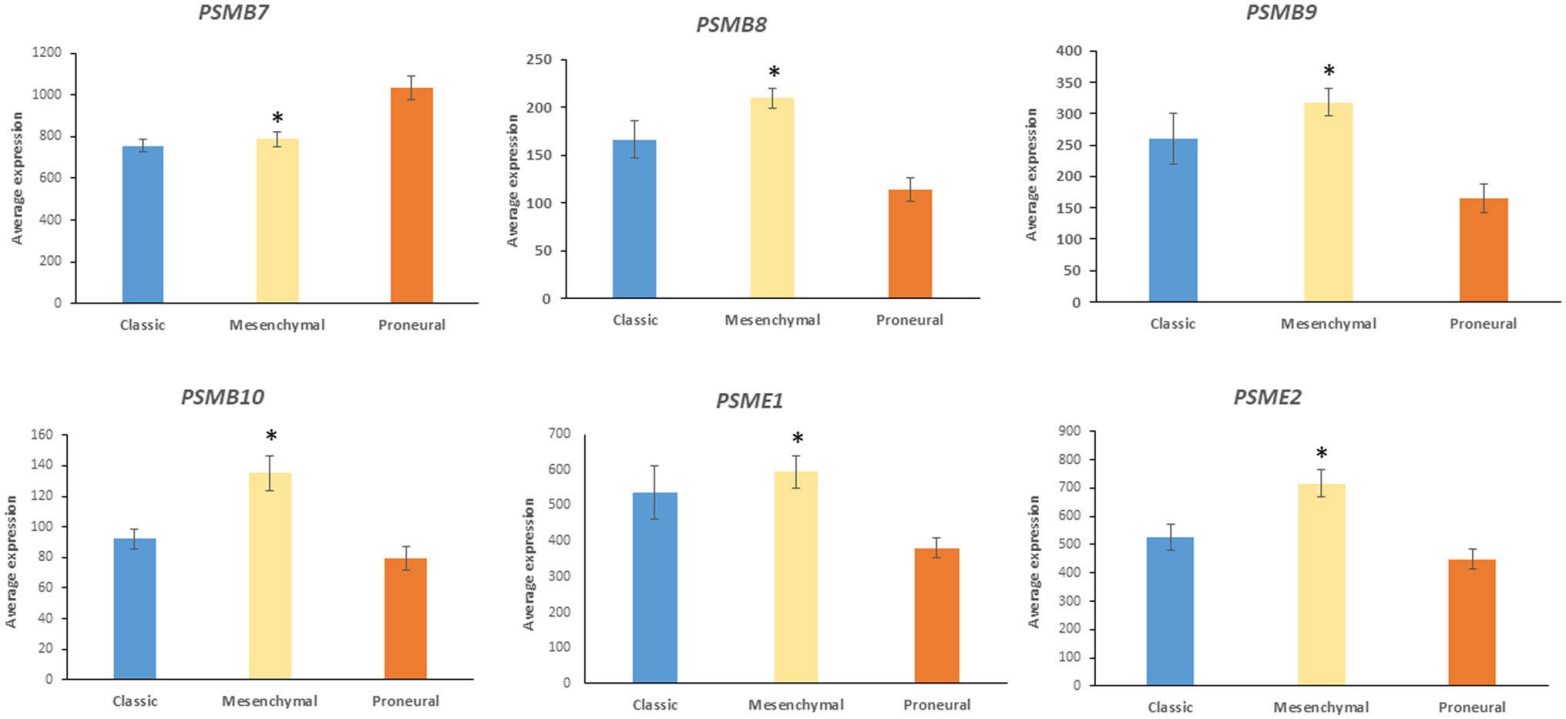
Gene expression of proteasome subunits in GBM subtypes. *PSMB7*, *F = *11.12, *p* = 7.03 × 10^−5^; *PSMB8***,** *F = *14.61, *p* = 5.74 × 10^−6^; *PSMB9***,** *F = *9.23, *p* = .08 × 10^−4^; *PSMB10,* *F = *10.61, *p* = 1.03e^−4^; *PSME1*, *F = *5.84, *p* = 4.64e^−3^; *PSMB2*, *F = *10.55, *p* = 1.08e^−4^). *By *t* test, genes coding for the immunoproteasome subunits, *PSMB8*, *PSMB9*, *PSMB10*, *PSEME1*, and *PSME2,* but not *PSMB7*, are significantly elevated (at *p < *0.001) in the mesenchymal group compared to the proneural group. *PSMB7* expression in the mesenchymal group is significantly lower than that of the proneural group

Expression of *PSMB7*, which encodes a catalytic subunit of the standard proteasome, was relatively increased in the proneural GBM group. Expression of *PSMB8*, *PSMB9*, and *PSMB10*, which encode catalytic subunits of the immunoproteasome, and the proteasome activator genes, *PSME1* and *PSME2*, was relatively increased in the mesenchymal GBM subtype (Fig. 18). In addition to acting as proteasome activators, the PSME1 and PSME2 proteins, also facilitate antigen presentation. Modulation of the ubiquitin proteasome system has been described as a mechanism by which EBV contributes to malignant transformation (Masucci 2004). Frisan and colleagues reported that the EBV latency membrane protein, LMP-1, is associated with IFN-induced increase in proteasome subunits LMP7, LMP2, and MECL1; these proteins are encoded by PSMB8, PSMB9, and PSMB10, respectively (Frisan et al. 1998). Zavala-Vega et al. have raised the issue of whether the EBV association with GBM is a causative factor in oncogenesis but this issue has not been resolved (Zavala-Vega et al. 2019).

The data of Fig. 18 suggest a shift in gene expression towards genes associated with subunits of the immunoproteasome in the mesenchymal group. Like *PSMB8*, *PSMB9*, and *PSMB10*, the genes encoding the immunoproteasome 11S proteins, *PSME1* and *PSME2* are induced by interferon-γ (Kohda et al. 1998). Since the immunoproteasome contributes to antigen processing (McCarthy and Weinberg 2015), the data are consistent with increased antigen presentation in the mesenchymal GBM subtype. The immunoproteasome has been proposed as a therapeutic target in cancers and in neurological diseases (Zerfas et al. 2020). This form of the proteasome generates peptides that combine with peptides of the major histocompatibility complex (MHC) class 1 for the purpose of antigen presentation (McCarthy and Weinberg 2015; Ferrington and Gregerson 2012). The immunoproteasome also processes virus derived peptides, but virus derived peptides may interfere with normal processing of MHC peptides by the immunoproteasome (Hewitt 2003; McCarthy and Weinberg 2015).

**Fig. 19:**
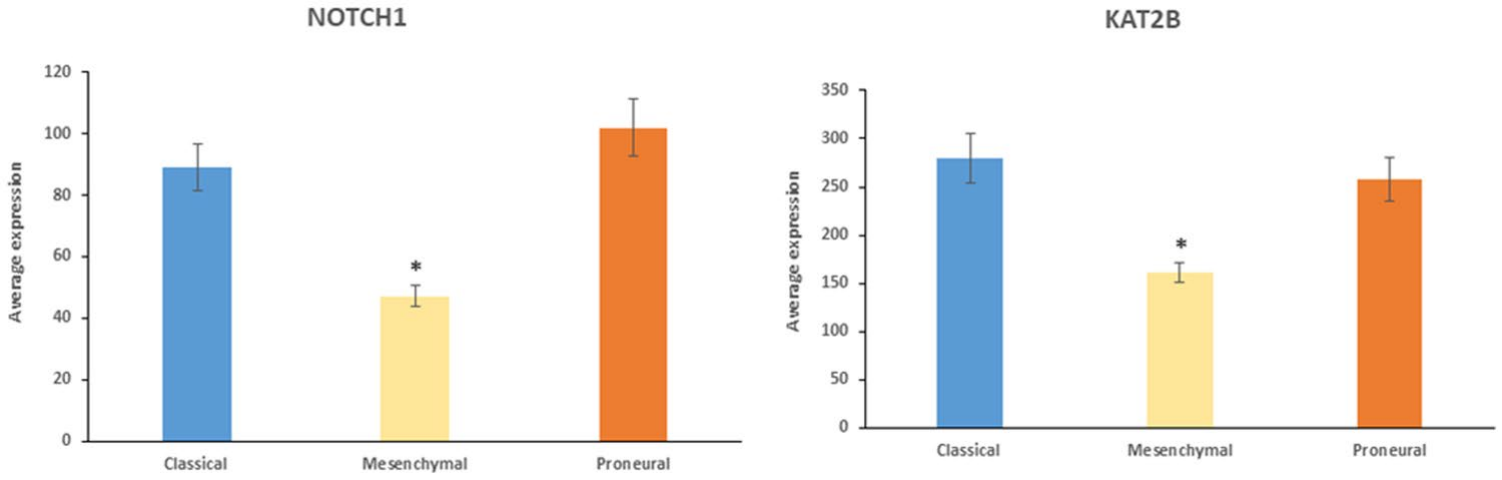
*NOTCH1* and *KAT2B* expression are elevated in classical and proneural GBM subtypes. *NOTCH1* (*F = *19.24, *p* = 2.73 10^−7^); *KAT2B* (*F = *10.96, *p* = 7.91 10^−5^). *By *t* test, mesenchymal average is significantly less (at *p < *0.001) than that of classical and proneural

The Notch signaling pathway was the second most significant pathway in the category of “*Regulation of stem cell differentiation*” and included the three genes, *HES1* (*F = *6.00, *p* = 4.07 × 10^−3^), *JAG1* (*F = *14.37, *p* = 6.78 × 10^−6^), and *NOTCH1* (*F = *19.24, *p* = 2.73 10^−7^). The *NOTCH1* gene encodes for the NOTCH1 receptor and *JAG1* encodes for the Notch ligand Jagged-1, while *HES1* encodes a Notch target gene. These data suggest the importance of the functionality of the Notch pathway in stem cell differentiation in at least one of the subtypes of GBM. Hai et al. 2018 reported that *NOTCH1* expression was higher in GBM than in non-tumor brain tissue and that *NOTCH1* expression was greater in classical and proneural GBM subtypes than in mesenchymal GBM, which is in agreement with our data from the Sun and TCGA datasets (Hai et al. 2018). NOTCH1 signaling and the interaction of the Notch and NF-kB pathways were shown to contribute to GBM growth and survival (Hai et al. 2018; Yi et al. 2019). While NOTCH1 may be a potential therapeutic target for various tumors (Guo et al. 2014), one or more of the HATS proteins, which also act as E3 ligases (Fig. 6) and transcription regulators of the NICD intracellular domain (NICD) transcription complex, may also be considered as therapeutic targets for GBM subtypes. The distribution of *NOTCH1* and *KAT2B* expression in the TCGA glioblastoma dataset is show in Fig. 19.

*In summary*, the major KEGG biological pathways associated with ‘*Regulation of stem cell differentiation*’ were the proteasome pathway (genes for proteasome subunits) and the Notch signaling pathway. Genes coding for the immunoproteasome and the HIPPO pathway were elevated in mesenchymal versus proneural GBM subtype, with intermediate expression in the classical GBM subtype.

**Fig. 20:**
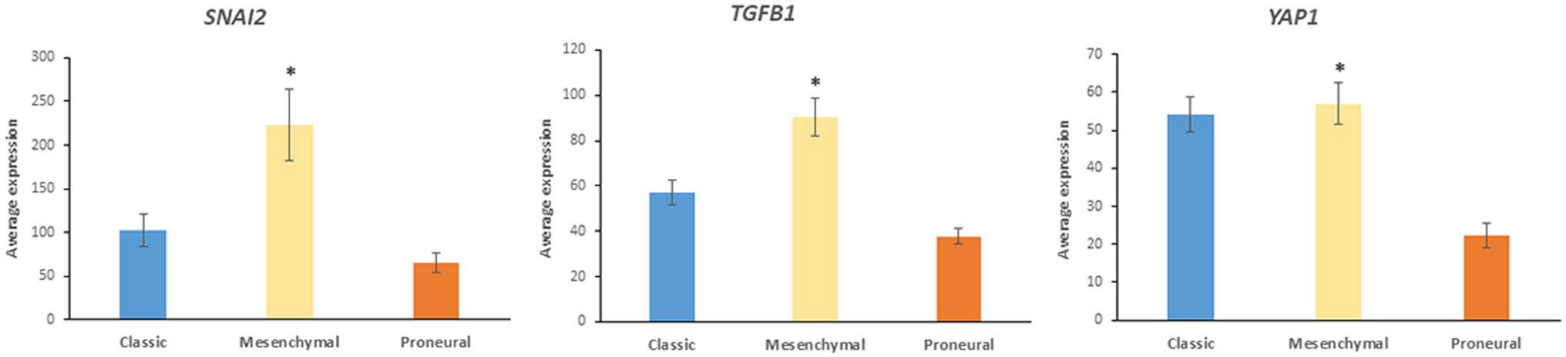
Hippo pathway gene expression was significantly elevated in the mesenchymal GBM subtype compared to proneural GBM subtype. *SNAI2* (*F = *8.195, *p* = 6.70 × 10^−4^); *TGFB1* (*F = *19.218, *p* = 2.77 × 10^−7^); *YAP1* (*F = *18.225, *p* = 5.21 × 10^−7^). *By *t* test means in mesenchymal GBM were significantly greater (*p < *0.0001) than that for each of the three genes in proneural GBM

Similar to the results with the Sun dataset, the pathway statistically most associated with “*Regulation of stem cell proliferation*” in the TGGA dataset was the Hippo signaling pathway (Table 11). In both cases, expression of three genes, *SNAI2*, *TGFB1*, and *YAP1*, contributed to the differential expression in GBM subtypes (Fig. 20). Expression of these three genes was significantly greater in the mesenchymal versus the proneural GBM subtype (*t = *3.50, *p < *0.001; *t = *5.68, *p < *0.0001; *t = *5.41, *p < *0.0001, respectively).

**Table 11:** Top KEGG pathways associated with “*Regulation of stem cell proliferation”* in the TGCA dataset

KEGG pathway over-represented	*p* value for pathway	Genes
Hippo signaling	1.13 × 10^*−*13^	*FZD3, SNAI2, TGFB1, YAP1*
Renal cell carcinoma	9.4 × 10^*−*6^	*HIF1A, TGFB1*
Proteogylcans in cancer	3.3 × 10^*−*5^	*FZD3, HIF1A, TGFB1*

### Differential Expression of Deubiquitinases (DUBs) in GBM Subtypes

The expression of several DUB genes was significantly elevated in the proneural GBM subtype (*USP11*, *F = *22.24, *p* = 4.38 × 10^*−*8^; *USP22*, *F = *14.79, *p* = 5.9 × 10^*−*6^; *USP7*, *F = *13.97, *p* = 8.98 × 10^*−*6^; *USP33*, *F = *7.57, *p* = 1.11 × 10^*−*3^). The gene encoding human dual E3 ligase and deubiquitinase *TNFAIP3* was significantly elevated in mesenchymal GBM subtype compared to classical and proneural GBM subtypes (*F = *19.77, *p* = 1.96 × 10^*−*7^). The *TNFAIP3* gene product A20 regulates NF-kB signaling in response to DNA damage and serves as a predictor of GBM patient survival and therapeutic target of GBM (Bredel et al. 2006).

### Upregulation of key UPS Genes Correlates with Protein Detection in Brain Tumor Tissues

Several UPS genes with elevated transcription in GBM in the Sun glioma dataset were reported in the literature with increased protein levels as well. These include *UBE2C* and *UBE2S* (Fig. 2). Elevated protein of UBE2C and UBE2S have been shown to occur in high-grade gliomas (Ma et al. 2016; Hu et al. 2021). These two genes encode E2 conjugase proteins involved in regulation of the APC/c complex during the cell cycle. As such these proteins would be good candidates as therapeutic targets in GBM. The E3 adaptor gene, *CDC20*, whose expression is elevated in GBM (Fig. 13), encodes another key regulator of the APC/c complex during the cell cycle. The CDC20 protein has been shown to be elevated in GBM stem cells (Gujar et al. 2016). Elevated expression of the genes *AURKA* and *TPX2* (Fig. 13) and their respective proteins have also been noted in high-grade gliomas and have been proposed as therapeutic targets (Barton et al. 2010; Samaras et al. 2009). AURKA and TPX2 also contribute to regulation of the cell cycle.

Pathways in addition to those involved in regulation of the cell cycle may be over-expressed in GBM. A role for SOCS proteins as biomarkers has been recently reviewed (Dai et al. 2022). The Sun dataset also shows elevated gene expression of the SOCS genes, *SOCS1*, *SOCS2*, and *SOCS3* in GBM (Fig. 8). Another protein elevated in gliomas compared to normal brain is the immunoproteasome protein, LMP7, encoded for by th*e gene PSMB8* (Min et al. 2019). We noted elevated expression of several immunoproteasome genes, including *PSMB8 and PSMB9*, in GBM (Fig. 14), particularly in the mesenchymal subtype (Fig. 18). The correlation of gene expression and protein levels can be further determined for other biological pathways that are dysregulated in GBM, including the Notch signaling pathway (Bazzoni and Bentivegna 2019).

### Ubiquitin Ligases and Their Adaptors Include Autophagy Related Genes

Autophagy is a mechanism for eliminating proteins and cellular organelles to maintain homeostasis under cellular stress (Torrisi et al. 2022). The role of the UPS in regulating autophagy has been reviewed recently (Chen et al. 2019; Kocaturk and Gozuacik 2018). However, the interaction of the UPS and autophagy in gliomas and the role of autophagy in cancer is still emerging (Yun and Lee 2018). We determined the list of differentially expressed genes in the heatmaps of E3 ligases and adaptors in the Sun and TCGA datasets associated with apoptosis, using the R2 genomics platform, and identified genes associated with the GO category of *autophagy*. Four genes encoding E3 ligases (HERC1, TRIM5, TRIM21, and RNF41) and three genes encoding E3 ligase adaptors (WDFY3, WDR41, WIPI1) were identified in the Sun dataset in the GO category of *autophagy*. Among the E3 ligases and adaptors in the TCGA GBM dataset, five genes encoding for E3 ligases (TRIM21, TRIM22, TRIM5, RNF5, and FYCO1) and four genes encoding E3 adaptors (WIPI1, ZBTB17, PLAA, and WDR6) were associated with autophagy.

**Fig. 21:**
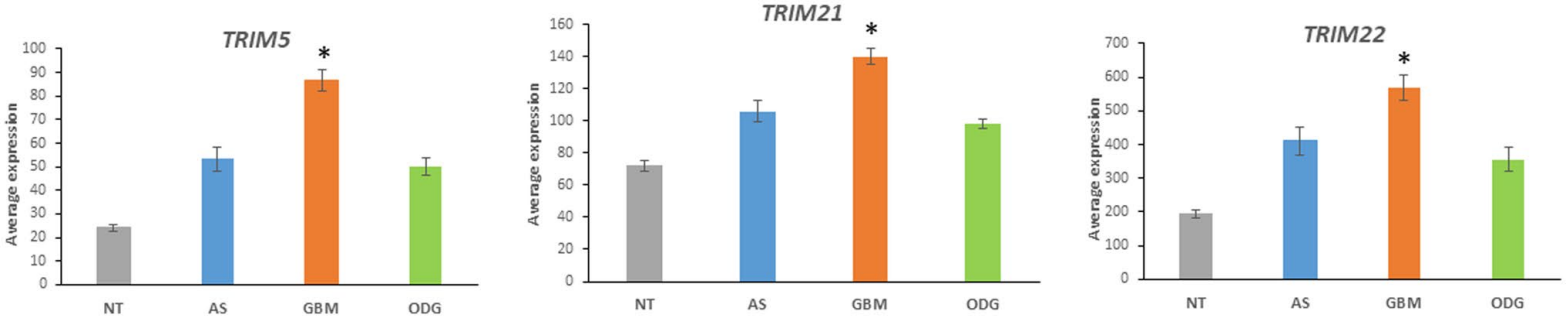
Increased expression of TRIM genes in glioblastoma (TRIM5 *F = *29.14, *p* = 2.78e^−15^; TRIM21 *F = *31.53e^−16^, *p* = 2.72e^−16^; TRIM22 *F = *13.68, *p* = 4.78e^−08^). *significantly different from all NT groups at *p < *0.0001, from ODG groups at least *p < *0.01, from AS groups at least *p < *0.05)

GO network analysis of the autophagy related genes among the E3 ligase and E3 adaptor genes above, showed that the GO pathways of “*Regulation of symbiont entry into host* and *regulation of viral entry into host”* were over-expressed. The E3 ligase genes contributing to these categories were *TRIM5*, *TRIM21*, and *TRIM22*. These genes were also associated with the GO category of “*Interferon stimulated genes”;* the role of type 1 and type 2 interferons in antiviral immune response is well documented (Kocaturk and Gozuacik 2018; Lee and Ashkar 2018). TRIM proteins have been reported to regulate autophagy (Mandell et al. 2014; Kimura et al. 2017) and TRIM5, TRIM 21, and TRIM22 are among the TRIM proteins associated with antiviral responses (Carthagena et al. 2009). Expression of these genes was greater in the gliomas (particularly in the GBM group) than in NT samples in the Sun dataset (Fig. 21). These data suggest the possibility that apoptosis can be induced in cells infected with viruses (Nainu et al. 2017) in GBM. TRIM ubiquitin ligases warrant further investigations as to their role in autophagy and GBM (Yun and Lee 2018).

## Summary and Conclusions

Our analysis of the Sun and TCGA glioma datsets identified significant changes in the expression of selected genes encoding E2 conjugases, E3 ligases and their adaptors, proteasome subunits, and DUBs. These transcriptional changes implicate major components of the UPS pathway in glioma pathogenesis and suggest specific UPS functions in AS, GBM, ODG, and different GBM subtypes. Over-expression of the genes encoding the ubiquitin conjugases, UBE2C and UBE2S, was noted in GBM.

**Fig. 22:**
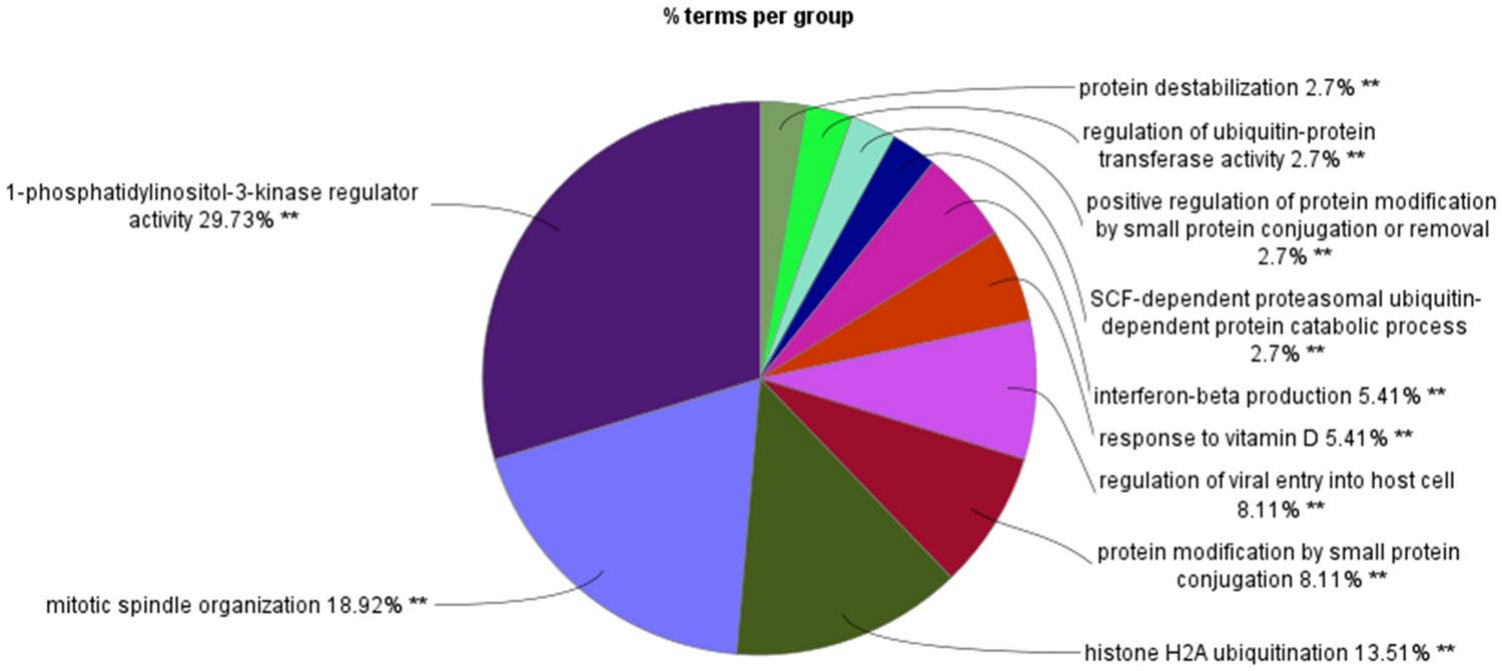
Cytoscape analysis of GO *biological processes* represented by differentially expressed E3 ligase and ligase adaptor genes in the Sun dataset

In addition to the UPS pathway, Cytoscape GO biological pathway analysis of the differentially expressed genes encoding E3 ligases and adaptors in the Sun dataset highlighted the biological processes PI-3 K regulation, mitotic spindle organization, histone ubiquitination, interferon beta production, and regulation of viral entry into host cells (Fig. 22).

The cluster analysis illustrated in the heatmaps of Figs. 3 and 7 suggest the probability of a single factor regulating the activity of numerous ubiquitin ligases. One biological pathway that regulates E3 ligases is neddylation. Protein conjugation of NEDD8 leads to the activation of many Cullin Ring ligases (Merlet et al. 2009). Inhibition of neddylation has anticancer effects by stimulating apoptosis and autophagy (Zhou et al. 2019). The neddylation inhibitor MLN4924, also known as pevonedistat, is currently being investigated as a therapeutic agent in cancer treatment (Zhao et al. 2014). In vitro studies, and a xenograft mouse model, suggest that MLN4924 is a promising therapeutic agent in GBM (Hua et al. 2015). We suggest that the neddylation inhibitor MLN4924 may be efficacious in the treatment of at least one of the GBM subtypes.

KEGG analysis, and Reactome analysis, of differentially expressed genes for E3 ubiquitin ligases, identified differential expression of genes associated with the Notch signaling pathway in AS, GBM, and ODG glioma and in GBM subtypes. Differentially expressed genes for E3 ubiquitin ligase adaptors showed over-expression of the *SOCS* (Suppressor of cytokine) signaling genes and genes of the Hippo signaling pathway in GBM. The Sun data showed upregulated HIPPO target genes via YAP/ TAZ/ TEAD-induced transcription in the GBM group, with an epigenetic regulatory role for E3 ubiquitin ligases and their adaptors. The selective transcriptional downregulation of ubiquitin ligase adaptors and PP2A phosphatase regulatory isoforms *PPP2R2C* and *PPP2R2D* in GBM versus AS, ODG, and non-tumor brain samples suggested an intricate role of the ubiquitination machinery on Hippo signaling. By regulating the dephosphorylation YAP/TAZ, protein phosphatase 2A can control nuclear entry of the transcriptional coactivators (Sarmasti Emami et al. 2020).

In GBM, the over-expression of genes encoding immunoproteasome subunits PSMB8 and PSMB9 emphasized the importance of the immunoproteasome for GBM biology. Several genes associated with the GO category of “*Regulation of viral entry into host cells*,” *TRIM5*, *TRIM21, TRIM22*, and *TRIM38* were over-expressed in GBM compared to ODG and non-tumor samples. These genes encode proteins involved in antigen processing and regulation of interferon mediated immune responses. These data suggest the immunoproteasome as a promising therapeutic target in GBM.

**Fig. 23:**
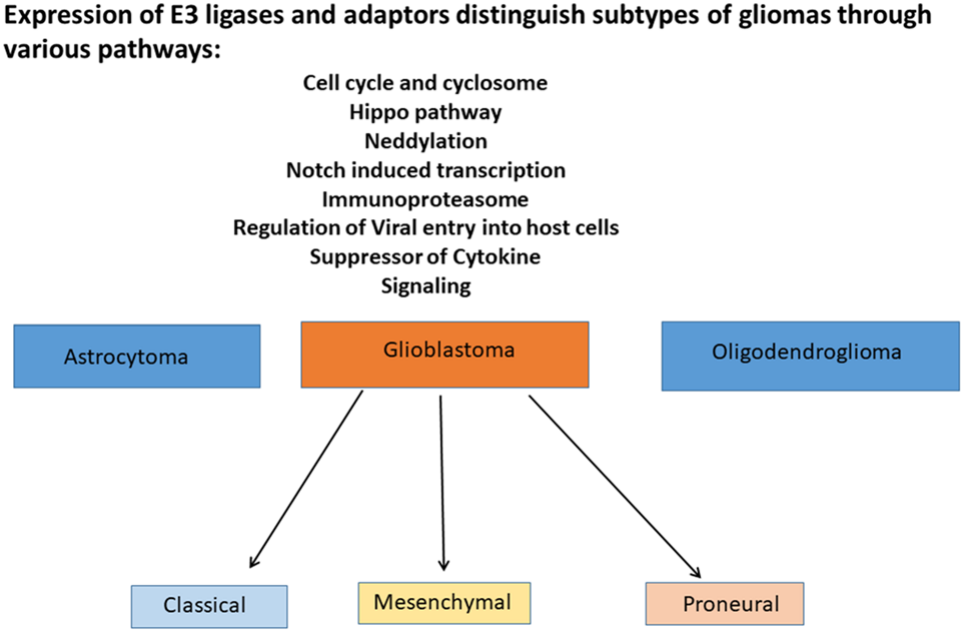
Biological pathways associated with differential expression of E3 ligases and adaptors distinguish gliomas of astrocytic and oligodendroglial origin

*In summary*, the major pathways associated with differential expression of E3 ligases and adaptors identified among gliomas in this study are shown in Fig. 23

The complex biological functions of the many UPS components remain largely unknown in glioma. Our results indicate that effective therapeutic targeting of components of the UPS should include considerations on the glioma and GBM subtype-specific differences in gene expression of UPS members and the effects thereof on major downstream signaling cascades.

## Data Availability

The data referred to in this manuscript are publicly available at the R2 Genomics Analysis and Visualization Platform **(**http://r2.amc.nl**)** and GSE4290.
